# Elucidation of Exosome Migration across the Blood-Brain Barrier Model In Vitro

**DOI:** 10.1007/s12195-016-0458-3

**Published:** 2016-07-07

**Authors:** Claire C. Chen, Linan Liu, Fengxia Ma, Chi W. Wong, Xuning E. Guo, Jenu V. Chacko, Henry P. Farhoodi, Shirley X. Zhang, Jan Zimak, Aude Ségaliny, Milad Riazifar, Victor Pham, Michelle A. Digman, Egest J. Pone, Weian Zhao

**Affiliations:** 1 Department of Pharmaceutical Sciences, Sue and Bill Gross Stem Cell Research Center, Chao Family Comprehensive Cancer Center and Edwards Life sciences Center for Advanced Cardiovascular Technology, 845 Health Sciences Road, University of California-Irvine, Irvine, California, 92697, USA; 2 Department of Biomedical Engineering, University of California-Irvine, Irvine, California, 92697, USA; 3 State Key Laboratory of Experimental Hematology, Institute of Hematology and Blood Disease Hospital, Chinese Academy of Medical Sciences and Peking Union Medical College, Tianjin 300020, China; 4 Department of Molecular Biology & Biochemistry, University of California-Irvine, Irvine, California, 92697, USA; 5 Laboratory for Fluorescence Dynamics, University of California-Irvine, California 92697, USA; 6 Centre for Bioactive Discovery in Health and Ageing, School of Science and Technology, University of New England, Armidale, New South Wales 2351, Australia

**Keywords:** Drug delivery, blood-brain barrier (BBB), exosome, humanized *Gaussia* luciferase (hGluc), stroke, inflammation, endocytosis, exocytosis, transcytosis

## Abstract

The delivery of therapeutics to the central nervous system (CNS) remains a major challenge in part due to the presence of the blood-brain barrier (BBB). Recently, cell-derived vesicles, particularly exosomes, have emerged as an attractive vehicle for targeting drugs to the brain, but whether or how they cross the BBB remains unclear. Here, we investigated the interactions between exosomes and brain microvascular endothelial cells (BMECs) *in vitro* under conditions that mimic the healthy and inflamed BBB *in vivo*. Transwell assays revealed that luciferase-carrying exosomes can cross a BMEC monolayer under stroke-like, inflamed conditions (TNF-α activated) but not under normal conditions. Confocal microscopy showed that exosomes are internalized by BMECs through endocytosis, co-localize with endosomes, in effect primarily utilizing the transcellular route of crossing. Together, these results indicate that cell-derived exosomes can cross the BBB model under stroke-like conditions *in vitro*. This study encourages further development of engineered exosomes as drug delivery vehicles or tracking tools for treating or monitoring neurological diseases.

## INTRODUCTION

Despite significant advances in drug delivery, a major challenge remains in delivering therapeutics effectively to the brain for the treatment of central nervous system (CNS) diseases, including trauma, stroke, autoimmune diseases, neurodegenerative diseases and tumors^1, 4, 35, 48, 67^. Drug delivery to the CNS is limited by the presence of the blood-brain barrier (BBB), a dynamic interface that restricts and controls the passage of substances between the peripheral vascular circulation and the CNS, thus serving to protect the CNS from harmful substances or overzealous immune respenses^3, 4, 35^. The BBB is composed of brain microvascular endothelial cells (BMECs), astrocytes, pericytes, the endothelial basement membrane, and adjacent neurons. The brain endothelial cells have a complex arrangement of tight junctions (TJs) and adherens junctions (AJs), which play key roles in regulating paracellular permeability^72^. These junctions prevent transport of most molecules except those normally used for homeostasis, including for nutrition or bidirectional hormonal communication and reflecting the changing properties of the BBB depending on conditions^4^.

While this complex interface protects the brain from harmful chemicals or toxins that may be present in systemic circulation, it also results in the inability of therapeutics to cross the BBB, with approximately 98% of small molecule pharmaceuticals and almost all of large molecule biologic drugs, including recombinant proteins, monoclonal antibodies, or gene-based medicines, failing to cross the BBB^40, 41^. However, under certain CNS disease states, the BBB is dysregulated or malfunctioned, which could itself be used as a passive mechanism for targeting therapeutics to the brain^52^. For example, under ischemic stroke and subsequent reperfusion condition caused by arterial embolism or thrombosis, the integrity of TJs of the BBB is compromised, leading to increase in paracellular permeability and allowing entry of both small and large molecules into the brain^35, 49^. And in autoimmune CNS diseases such as multiple sclerosis (MS), lymphocytes can enter sclerotic lesions, though the sequence of pathological events and immune infiltration remain to be fully elucidated^8^. Administration of human basic fibroblast growth factor (bFGF), an endogenous neurotrophin that does not cross the BBB^71^, exerted a neuroprotective effects in the post ischemic brain^53^, implying that it could diffuse across the compromised BBB. Moreover, the so-called enhanced permeability and retention (EPR) effect has also been used to transport anticancer drugs using nanocarriers such as nanoparticles and liposomes that can accumulate and passively extravasate into the tumor vasculature^42, 65^. But although particulate drug carriers such as dendrimers^27^, nanoparticles^43^ and liposomes^64^ have been tested for drug delivery across the BBB, they remain not widely used in the clinic considering their immunogenicity, limited half-life *in vivo*, and, importantly, relative lack of specificity and efficacy in crossing the BBB^17, 67^.

Cell-based medications are a newer class of drug delivery for CNS diseases ^5, 30^. Stem cells, such as mesenchymal stem (or stromal) cells (MSC), are known to mobilize from the bone marrow or fat deposits and migrate and home to sites of injury^54^, including CNS injury^5^, where they presumably exert protective and recovery effects via numerous paracrine factors^28^. Administration of human bone marrow MSC can enhance recovery from CNS injuries^11, 30^, by their secretion of exosomes, growth factors, cytokines, and other paracrine factors ^5, 74^. More recently, natural cell-derived vesicles collectively termed extracellular vesicles (EVs) have been investigated as a new class of carriers of drugs, nucleic acids and diagnostic reagents^25^, including for CNS diseases^73^. In particular, exosomes that originate from intracellular multivesicular bodies (MVBs) and represent a major subtype of EVs have recently been reported to be involved in a variety of activities in the normal and pathological CNS^12, 16, 21^, thus rendering them potentially attractive vehicles for delivering agents across the BBB.

There are several reasons for the recent resurgence of activity in testing EVs as therapeutics and as vectors for therapeutic delivery, including for CNS diseases. Although small relative to cells, EVs host a complex mixture of surface receptors and intravesicular cargo, including proteins and nucleic acids, that may synergize to enhance therapeutic efficacy compared to isolated factors^61, 68^. EVs have been found to mediate, in part, the curative effects of cell-based therapies, especially for stem cells ^23^. In particular, exosomes derived from MSCs have been reported to exhibit neuroprotective effects and promote tissue repair in CNS injury models^73, 74^. Similar to MSC, hematopoietic stem cells (HSC), are known to increase their activity in bone marrow as well as enter peripheral circulation in response to infection or injury^66^. Uptake of exosomes purified from HSC and injected into the cerebellum by Purkinje neurons was reported in a cre-reporter model, though whether exosomes could cross the BBB was not investigated^46^. Other cell types that possibly secrete exosomes capable of bypassing the BBB are immune cells and CNS cells themselves, including BMECs, neuronal types, astrocytes, microglia and their progenitors. BMEC-derived exosomes were reported to deliver the anticancer drug doxorubicin across the BBB in a glioma model^75^. Exosomes from dendritic cells (DCs) delivered small interfering RNA (siRNA) to treat Alzheimer’s disease to the mouse brain, suggesting they may cross the BBB and deliver siRNA cargos into target cells for specific gene knockdown^2^. Exosomes derived from a mouse lymphoma cell line could deliver curcumin across the BBB to microglial cells via intranasal administration to attenuated brain inflammation and autoimmune responses in experimental autoimmune encephalomyelitis (EAE)^77^, whereas curcumin-primed exosomes ameliorated oxidative stress and tightened AJs and TJs induced by hyperhomocysteinemia, leading to a reduction of permeability^21^.

Thus, considering that exosomes have recently been found to play key roles in CNS homeostasis, pathology and subsequent recovery, their natural favorable characteristics (lack of immunogenicity and prolonged half-life) could be combined with additional bioengineering approaches to enhance their biodistribution, including increased ability to bypass the BBB^50, 73^. Nevertheless, although published data suggested that exosomes could deliver therapeutics to the brain, the mechanisms of interaction between exosomes and the BBB remain elusive. Therefore, as a step towards efficient therapeutic delivery to the brain using exosomes, this study aims to elucidate whether and how exosomes bypass the BBB. Using engineered HEK 293T-derived exosomes and *in vitro* BMEC monolayers as a model system; we demonstrated that cell-derived exosomes can cross the BBB model via mostly active BMEC endocytosis primarily utilizing the transcellular route of crossing under stroke-like conditions *in vitro*.

## MATERIALS AND METHODS

### Cell lines and cell culture

HEK293T cells (293T, GenTarget) were cultured in high-glucose Dulbecco’s modified Eagle’s medium (DMEM, Corning Cellgro) supplemented with 10% fetal bovine serum (FBS, Atlanta Biologicals), 100 U/mL penicillin, and 100 µg/mL streptomycin (Life Technologies). Brain microvascular endothelial cells (BMECs) were obtained from American Type Culture Collection (ATCC) and expanded in endothelial cell growth medium (Lonza) supplemented with SingleQuot Kit Supplements and growth factors (Lonza). All cultures were maintained at 37°C with 5% CO_2_.

### Generation of constructs and lentiviral transduction

The following constructs were used in this study: hGluc, hGluc-Lactadherin and hGluc-Lactadherin-GFP (Fig. S1a). The sequences of interest were obtained from pCMV-MFGE8-GFP (Origene) and LV-hGluc^31^. These were cloned into a lentiviral transfer vector LV-PL4 (GenTarget) using overlap PCR^56^. Lentivirus was packaged and 293T cells were then transduced and selected as previously described^31^. Engineered cells were visualized for transduction efficiency using fluorescence microscopy (Nikon).

### Exosome purification

To purify exosomes, conditioned medium was collected from 293T cells cultured for 48 hours in DMEM supplemented with 10% exosome-depleted FBS. First FBS was depleted of bovine exosomes by centrifugation at 100,000*g* at 4°C for 18 hours, followed by filtration through a 0.22 µm filter (Millipore). The cell supernatant was centrifuged at 300*g* for 10 minutes and 16,500*g* for 20 minutes at 4 °C to remove cell debris and microvesicles (MVs), respectively. Next, exosomes were pelleted by ultra-centrifugation at 120,000*g* using Beckman Coulter Optima L-80 XP ultra-centrifuge (Beckman Coulter) for 2.5 hours at 4°C and washed once in phosphate-buffered saline (PBS). Exosomes were resuspended in PBS or in lysis buffer for experimental analysis.

### Nanoparticle tracking analysis

Nanoparticle tracking analysis (NTA) was performed using the NanoSight NS300 system (Malvern). Samples were diluted 1:5000 with PBS, yielding particle concentrations between 3 × 10^8^ and 6 × 10^8^ particles per milliliter. The size of the exosomes was determined based on both light scattering and Brownian motion, and calculated using the Stokes-Einstein equation with NTA 3.0 analytical software (Malvern). The scattering mode was used for NTA, and both acquisition and analysis settings were kept constant for all samples. Each experiment was carried out in triplicate.

### Flow cytometry

Flow cytometric analysis was performed on exosomes immobilized on beads (Dynabeads 4.5 µm in diameter) bearing anti-CD63 mAb. The purified exosomes were incubated with Dynabeads overnight at 4 C with gentle agitation according to manufacturer’s protocol (Life Technologies). After three washes in PBS with 1% exosome-depleted FBS and 0.1% bovine serum albumin (BSA, Sigma-Aldrich), exosomes captured on beads were stained with PE conjugated CD9, CD63, or CD81 antibody, or isotype control (BD Pharmingen); beads without any antibodies were also used as an additional control. All flow cytometry data were collected on a BD LSRII flow cytometer (BD Bioscience) and analyzed with FlowJo software (FlowJo).

### Immunoblotting

293T cells expressing hGluc, hGluc-Lactadherin, hGluc-Lactadherin-GFP were washed in PBS and lysed in radioimmunoprecipitation assay (RIPA) buffer (Cell Signaling) with protease inhibitors (Sigma-Aldrich). Protein concentration was determined by bicinchoninic assay (BCA) (Thermo Fisher Scientific). The total exosome or cell protein lysate from each sample was loaded on a 4–15% SDS polyacrylamide gel (Bio-Rad) and transferred onto polyvinylidene fluoride membranes (Bio-Rad). Membranes were blocked in 5% milk or 5% BSA in Tris-Buffered Saline (Thermo Fisher Scientific) with 0.1% Tween-20 (Sigma-Aldrich) for 1 hour and incubated with anti-Gluc (1:1000, Nanolight), followed by binding of goat anti-rabbit IgG horseradish peroxidase (1:10,000, Santa Cruz Biotechnology). Bands were visualized using an enhanced chemiluminescence (ECL) kit (Thermo Fisher Scientific).

For exosome characterization, the total exosomal protein content isolated from the conditioned medium was quantified using BCA as above. The primary antibodies used in this study were anti-CD63 (1:1000, System Bioscience), anti-CD9 (1:1000, System Bioscience), anti-CD81 (1:1000, System Bioscience), anti-Gluc (1:1000, Nanolight), and anti-β-actin (1:2000, Abcam).

### Exosome labeling

Purified exosomes were labeled with PKH67 or PKH26 (0.5 µg/µL in PBS) Fluorescent Cell Linker Kit for General Cell Membrane Labeling (Sigma-Aldrich) according to the manufacturer’s protocol. As a control, PBS buffer alone was stained with PKH dyes. After staining, samples were washed three times with PBS, and fluorescently-labeled exosomes were concentrated using 300 kDa Vivaspin filters (Sartorius Stedim Biotech), as previously described^26^.

### In vitro BBB model and permeability assays

BMECs were grown on a type I collagen (BD Biosciences)-coated 6.5 mm transwell culture inserts with pore size of 0.45 µm (Corning Life Sciences) for 48 hours until a confluent monolayer was established. For BMEC activation, cells were treated with TNF-α (50 ng/mL, BD Biosciences) for 6 hours^29^. BMEC monolayer permeability was studied using Fluorescein isothiocyanate (FITC)-dextran (1 mg/mL) (Sigma-Aldrich), which was added to the upper chamber (luminal) and aliquots from the lower chamber (abluminal) were measured for their fluorescence intensity using a Biotek Synergy HT microplate reader (excitation at 485 nm and emission at 520 nm).

### In vitro bioluminescence assays

BMECs were grown in a transwell insert and characterized as mentioned above. Prior to addition of exosomes and their controls, cultures were stimulated with TNF-α for 6 hours and then washed, as previously described^29^. Untreated BMEC monolayers were used as an additional control. PKH67-labeled hGluc-Lact exosomes (10 µg, determined by protein content as previously described^24^) or controls (native exosomes or PBS) were then added to luminal chamber and incubated for 6 hours, 12 hours, 18 hours, and 24 hours at 37 °C, as indicated. Conditioned medium containing exosomes collected from luminal and abluminal chambers of transwell at different time points were plated in triplicate into a white 96-well luminometer plate. 25 µM (final concentration) of *Gaussia* luciferase substrate coelenterazine (CTZ, Nanolight) was added and bioluminescence activity was measured immediately using an IVIS Lumina (Caliper LifeSciences).

### Immunocytochemistry

BMECs were washed with PBS, fixed with 2% paraformaldehyde (PFA, Sigma-Aldrich) in PBS for 10 minutes at room temperature, and permeabilized with PBS containing 0.1% Triton X-100 (Sigma-Aldrich) for 10 minutes. After blocking with 1% normal donkey serum (Sigma-Aldrich) and 1% BSA in PBS containing 0.1% Triton X-100 (Sigma-Aldrich) for 1 hour, cells were subsequently incubated with anti-zonula occludens 1 (anti-ZO-1, 1:200, Life Technologies), anti-claudin-5 (1:50, Life Technologies), and anti-VE-cadherin (1:100, Santa Cruz Biotechnology) overnight at 4 °C. The BMECs were then washed with PBS and incubated with appropriate Alexa Fluor 594 donkey anti-rabbit IgG, Alexa Fluor 488 donkey anti-mouse IgG, or Alexa Fluor 594 donkey anti-goat IgG secondary antibodies (1:500, Jackson ImmunoResearch) for 1 hour at room temperature. To stain cell nuclei, cells were incubated in 4’,6-diamidino-2-phenylindole (DAPI, Life Technologies) at 1:300 dilution in PBS at room temperature for 5 minutes.

### In vitro confocal fluorescent imaging for exosome uptake and co-endocytosed localization

BMECs were grown on the coverglass coated with collagen I as described previously and stimulated with TNF-α for 6 hours. In order to image the time course of exosome uptake in BMECs, 24 hours prior to imaging, Deep Red Plasma Membrane Stain CellMask (Life Technologies) was used to label BMECs according to the manufacture protocol. Briefly, BMECs were incubated with CellMask in PBS for 5–10 minutes at 37 °C and then washed three times. PKH67-labeled exosomes (20 µg) or the control PBS stained with PKH67 were then added to BMECs and incubated for 1 hour, 3 hours, 6 hours, 12 hours, 18 hours, and 24 hours at 37 °C. All cells were then fixed with 2% PFA and nuclei were counterstained with DAPI. Image analysis was performed using a Zeiss LSM710 Multiphoton/Confocal microscope (Zeiss). The middle Z plane of the cell was imaged to ensure the elite imaging position. All the images were analyzed with Image J software (http://imagej.nih.gov/, NIH). For quantification, briefly, the outline of each cell (*n* > 30) was drawn referring to the cell membrane labeling (i.e., CellMask). The fluorescence intensity of intracellular exosomes that were specifically associated with the cells was then calculated. Triplicated samples were used for the analysis.

In order to identify the colocalization of endosomes and exosomes, BMECs grown on collagen I-coated coverglass were incubated with 10 µg/mL cholera toxin B subunit-biotin (CtxB, Sigma-Aldrich), following by Alexa Fluor 594 streptavidin (Jackson ImmunoResearch) conjugation, or 150 µg /mL transferrin-Texas Red (Tfn-Texas Red, Life Technologies) for 30 minutes at 37°C as previously described^55^. PKH67-labeled exosomes were subsequently incubated with cells for 1 hour or 3 hours. After incubation, excessive exosomes were washed three times with PBS. Cells were fixed with 2% PFA and nuclei were counterstained with DAPI. A Zeiss 63×1.4NA objective was used for the colocalization experiments. The images were then quantified using Image J Coloc2 plugin, which estimates the overlap coefficients in dual-color confocal images^32^. Colocalization of exosomes and Tfn/CtxB was indicated as yellow (green + red) pixels in the overlay images.

### In vitro cell viability and toxicity assays

BMECs were seeded at 20,000 cells/well in 96-well plates. Cells were treated with the indicated concentrations of endocytosis inhibitors or vehicle for 30 minutes, or with TNF-α or vehicle for 6 hours, to assess the cellular toxicity. Cell viability was determined using the 2,3-bis[2- Methoxy-4-nitro-5-sulfophenyl]-2H-tetrazolium-5-carboxyanilide inner salt (XTT) assay (ATCC) according to manufacture’s protocol. Briefly, the XTT reagent was added to each well, incubated for 2–4 hours at 37°C, and absorbance was measured at 450 nm with a reference wavelength 680 nm using a Biotek Synergy HT microplate reader. All samples were assayed in triplicate. The cytotoxicity of each inhibitor was compared to untreated controls.

### Exosome uptake and crossing inhibition studies

To further study the mechanisms of exosome uptake by BMEC monolayer, BMECs seeded on coverglass were pretreated with the following pharmacological inhibitors at their indicated concentrations: chlorpromazine (CPZ), cytochalasin D, amiloride, methyl- β -cyclodextrin (MβCD), filipin III, or nystatin for 30 minutes at 37°C before exosomes were added. The specificity and effective concentrations of endocytosis inhibitors were evaluated by measuring their effect on the markers for specific endocytic pathways. Specifically, Texas Red-transferrin (150 µg /mL) was used as a marker for clathrin-mediated endocytosis (CME), Alexa Fluor 594-CtxB (10 µg/mL) for lipid raft (caveolae)-mediated endocytosis, and Alexa Fluor 594-dextran (1 mg/mL) for macropinocytosis were used in this study. Inhibition was assessed by exosome uptake assay using confocal microscopy and image analysis. CellMask (Green Plasma Membrane Stain, Life Technologies)-labeled BMECs were then incubated with PKH26-labeled exosomes for 1 hour. After incubation, cells were washed with PBS, followed by fixation in PFA. Images were taken using a fluorescent microscopy (Nikon) and were analyzed with Image J software. To quantitatively measure the effects of above inhibitors on exosome crossing the *in vitro* BMEC monolayer in the transwell assay, the BMECs were pretreated with inhibitors prior to addition of exosomes, and conditioned medium from luminal and abluminal chambers were collected for bioluminescence analysis as described above.

### Statistical analysis

Data were presented as means ± SEM. Statistical differences were determined using unpaired Student’s *t* test when comparing between 2 independent groups, and oneway ANOVA with Student-Newman-Keuls (SNK) post-hoc test when comparing across 3 or more independent groups. *P* < 0.05 was considered significant.

## RESULTS AND DISCUSSION

### Exosome preparation, characterization and labeling

In order to monitor their distribution across BMEC monolayers *in vitro*, the exosomes were labeled with hGluc. hGluc was fused with lactadherin, which can be bound to cell membrane phophatidylserines (PS) and is also highly enriched on the outer leaflet of exosomal membrane^20, 56^. 293T cells were transduced with lentivirus expressing each free hGluc (hGluc 293T), hGluc-Lactadherin without (hGluc-Lact 293T) and with (hGluc-Lact-GFP) a Green Fluorescent Protein (GFP) tag used to monitor transduction efficiency (Fig. S1a). A high *Gaussia* luciferase expression was observed from the cell lysates of engineered 293T cells expressing hGluc-Lact (Fig. S1b, lane 2) and hGluc-Lact-GFP (Fig. S1b, lane 3) fusion proteins but not their counterpart (free hGluc alone, Fig. S1b, lane 1). These results indicate that engineered cells correctly expressed the membrane-targeted fused proteins. Moreover, the tagged hGluc proteins (i.e., hGluc-Lact and hGluc-Lact-GFP) were observed to associate with the cell membranes while free hGluc was mostly secreted into the conditioned medium (free hGluc expression in cells = 6.8%-7.8% of tagged hGluc expression in cells, Fig. S1b and S1c).

**Fig. 1:**
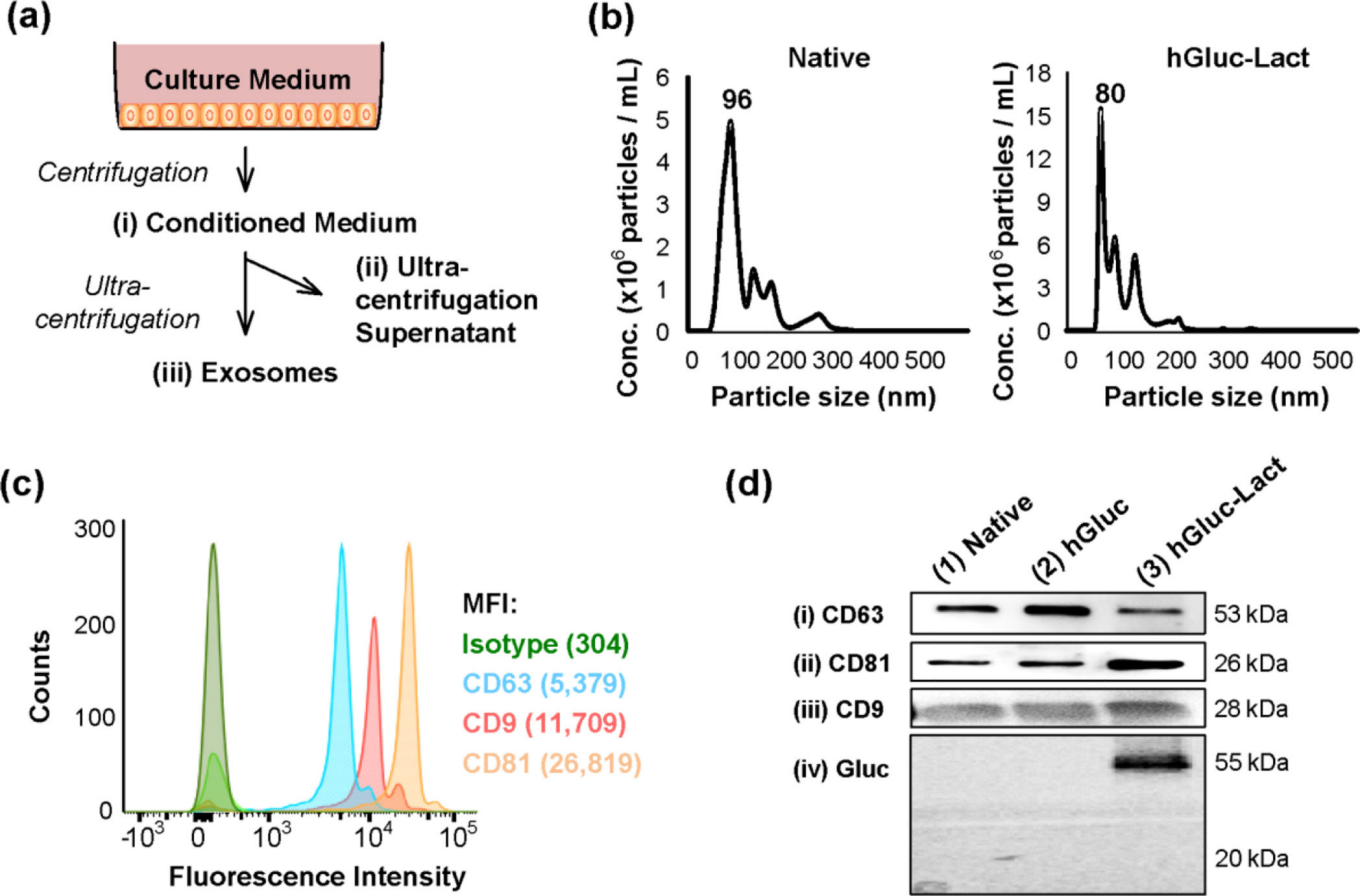
Exosome engineering and characterization (a) Schematic depiction of the isolation protocol for exosomes. (b) Size distribution of native and hGluc-Lact exosomes measured by nanoparticle tracking analysis (NTA). (c) Flow cytometry detection of exosome characterization. Exosomes were incubated with anti-CD63 Dynabeads and immunostained with exosomal surface markers (CD9, CD63, and CD81). Green histogram is the isotype control. Data were quantified and expressed as MFI (mean fluorescence intensity). (d) Western blot analysis of the marker proteins (i) CD63, (ii) CD81, and (iii) CD9 and (iv) Gluc on exosomes, respectively. Exosomes were purified from conditioned medium and characterized using western blot for the presence of typical exosomal markers CD63, CD81, and CD9. Additionally, Gluc detection by immunoblot of purified exosomes showed that hGluc-Lact fusion protein bound to the exosomal membranes.

Exosomes were then collected by ultra-centrifugation from conditioned medium of native and engineered 293T cells (Fig. 1a) and were characterized by NTA and biochemical analysis. The size distribution of purified exosomes was analyzed using NanoSight NS300 nanoparticle tracking analysis (NTA). NTA showed a similar size distribution profile for native (96.3 ± 5.4 nm) and hGluc-Lact exosomes (80.3 ± 2.0 nm) (Fig. 1b). Thus, our exosome preparations have similar size distribution as reported literature^24^, also suggesting that the modification with the expressed protein tags did not affect the physical properties of exosomes. Next, exosomes were characterized for expression of several typically prominent markers^59^. Exosomes were captured on beads coated with anti-CD63, a tetraspanin that is highly enriched in late endosomes, lysosomes and exosomes^44^, analyzed by flow cytometry after immunostaining with exosomal surface markers anti-CD9, anti-CD63, and anti-CD81 (Fig. 1c). Consistent with the literature^33, 59, 61^, our prepared exosomes are positive for CD9, CD63 and CD81. Western blotting further confirmed the presence of exosomal proteins CD9, CD81 and CD63 (Fig. 1d, lane 1). Next we examined whether hGluc-Lact was expressed on exosomes; indeed immunoblotting data showed that hGluc could only be detected on hGluc-Lact-labeled exosomes (hGluc-Lact exosomes) but not on hGluc alone counterparts, indicating that hGluc-Lact bound to exosome membranes with high specificity (Fig. 1d). Remarkably, the presence of exosomal biomarkers CD9, CD81 and CD63 was confirmed on engineered exosomes, without being affected by lentiviral transduction (Fig. 1d, lanes 2 and 3).

**Fig. 2:**
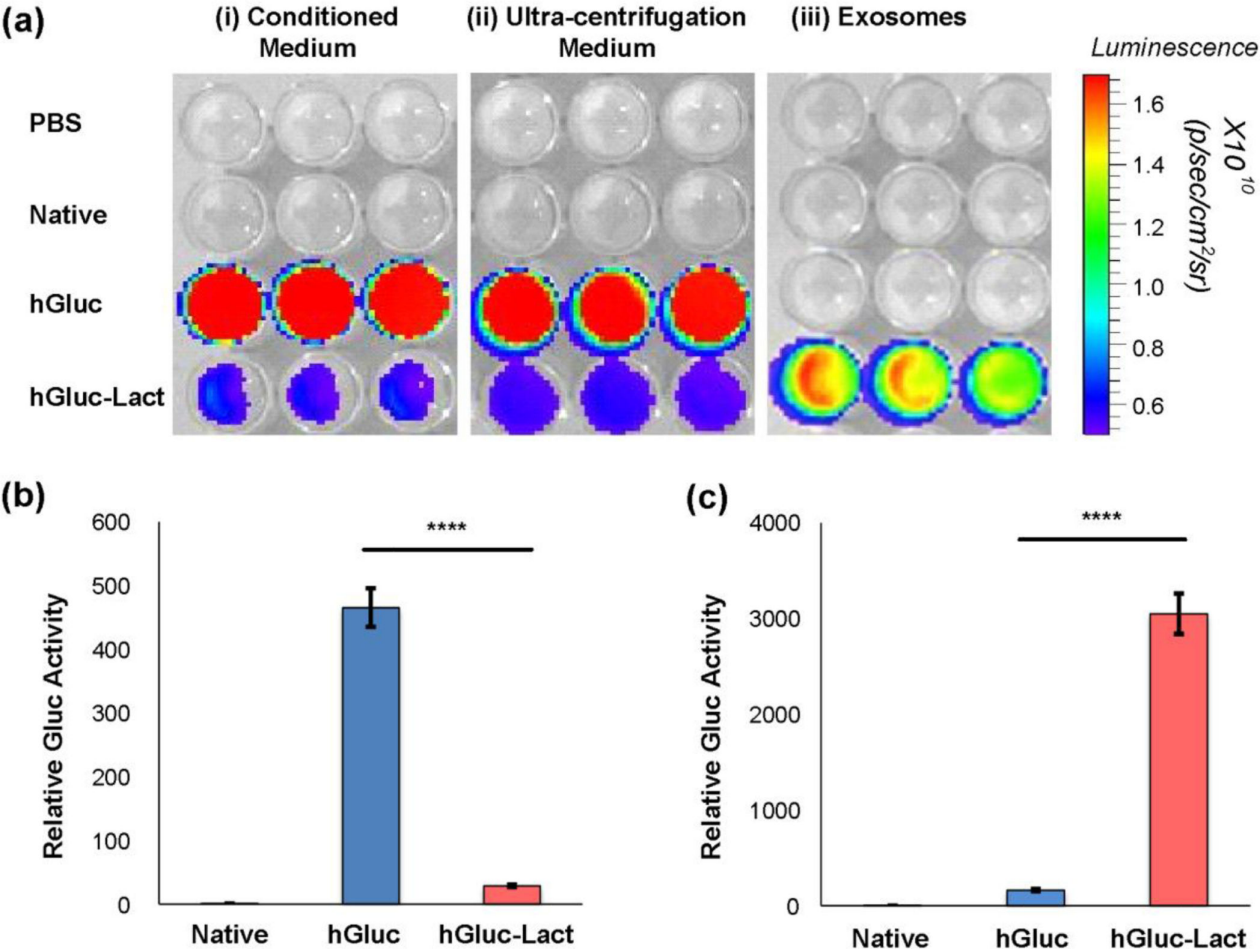
Validation of hGluc-labeled exosomes using *in vitro* bioluminescence Assays (a) *In vitro* bioluminescence assay of conditioned medium, ultra-centrifugation supernatant and exosomes. (i) Conditioned medium collected from 293T cells, (ii) supernatant collected after serial steps of ultra-centrifugation and (iii) exosomes purified from 293T cells through ultra-centrifugation were diluted in PBS. CTZ was then added at a final concentration of 25 µM. *Gaussia* luciferase (hGluc) activity was measured using IVIS Lumina (exposure time 0.5s). (b) hGluc activity was significantly higher in conditioned medium. Error bar: mean ± SEM. ****P < 0.0001. (c) After ultra-centrifugation, hGluc activity was mainly detected in hGluc-Lact exosomes, indicating hGluc was enriched on exosomes. Error bar: mean ± SEM. ****P < 0.0001.

A bioluminescence assay was then performed to confirm the presence of active luciferase on exosomes as well as to verify that the reporter is enriched on exosomes (Fig. 2a). Addition of luciferase substrate CTZ creates a bioluminescent signal whose intensity is proportional to the luciferase activity in the wells^58^. First, wells containing conditioned medium and its ultra-centrifugation supernatant from native 293T cells, hGluc 293T and hGluc-Lact 293T were measured for bioluminescent signals (Fig. 2a). Stronger signals in conditioned medium from hGluc 293T than those from hGluc-Lact 293T conditioned medium indicated that non-fused hGluc was secreted and was free from cells. Supernatant was separated from exosomes by ultra-centrifugation, and the bioluminescent signals in supernatant were of similar intensity to those from conditioned medium, confirming that free active hGluc was secreted as expected. Only isolated exosomes from hGluc-Lact 293T showed bioluminescence, indicating the lactadherin fused to hGluc kept the luciferase bound to the exosomes and could thus serve as a reporter for exosome spatial distribution in our subsequent studies. Moreover, quantitative analysis of bioluminescence from hGluc conditioned medium showed an approximately 10-fold greater signal than that from the hGluc-Lact conditioned medium (*P* < 0.0001, Fig. 2b). Isolated hGluc-Lact exosomes showed a much higher signal (> 100-fold, *P* < 0.0001, Fig. 2c) compared to exosomes derived from hGluc 293T, providing further evidence for successful production of exosome-bound luciferase reporter.

The stability of exosomes was analyzed at 37°C, 25°C, and 4°C for 24 hours, using NanoSight analysis to evaluate whether or not temperature altered their size distribution profiles (Fig. S2a). No significant difference was observed in exosome size among the three groups. In agreement with NTA, *in vitro* bioluminescence assays also revealed that the luciferase activity of exosomes was not affected by incubation at different temperatures for 24 hours (Fig. S2b). Similar to previous reports^18^, the exosomes used in our present studies are highly stable under the conditions of the assays performed throughout these studies.

### In vitro BBB model

**Fig. 3:**
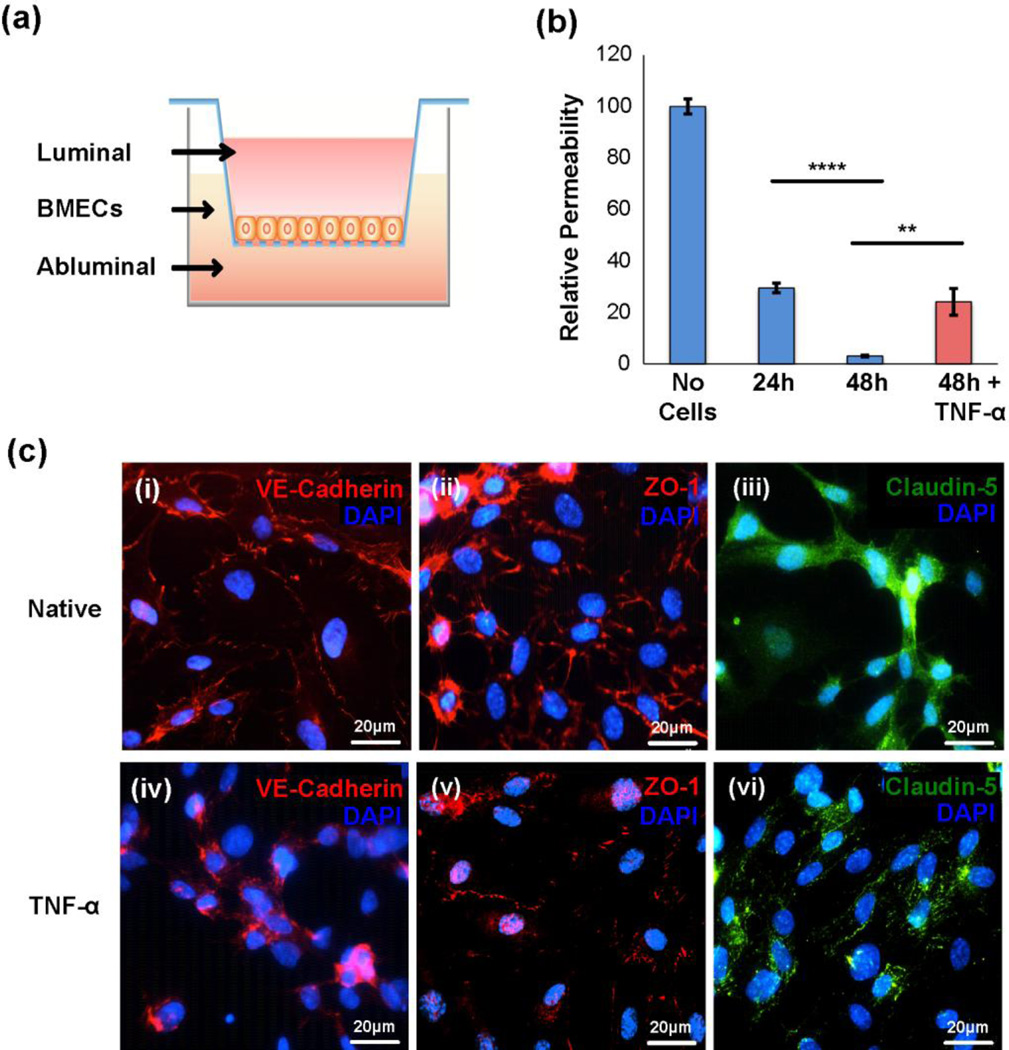
*In vitro* model of the BBB using BMEC monolayer indicated that stroke-like conditions increased its permeabilit *y* (a) The schematic representation of the *in vitro* model of BBB. (b) A BMECs monolayer was grown for 24 or 48 hours in a transwell insert, and then treated with TNF-α for 6 hours. Permeability was measured using FITC-dextran. BMECs formed a low permeability barrier after 48 hours, and permeability was increased significantly under TNF-α condition. Values represent as means ± SEM of relative ratio normalized to no cell control, set as 100%. **P < 0.01 and ****P < 0.0001 (c) Activation of BMECs with TNF-α regulates tight junction and adherens junction protein expressions in BMEC monolayer on coverglass. Immunofluorescence of VE-cadherin, ZO-1, and Claudin-5 showed that their expression levels were dramatically down-regulated after TNF-α treatment. DAPI was used for staining nuclei. Scale bar: 20 µm.

BMEC monolayers were cultured on collagen I-coated transwell insert or coverglass to mimic *in vitro* the BBB, allowing us to investigate the mechanisms involved in exosome interaction with the BBB and its potential bypass. The disruption of the BBB has been described as a crucial step of the neuroinflammatory response in cerebral ischaemia, and the junctional permeability of the BBB is controlled in large part by cytokines and other CNS factors^35^. With this understanding, we investigated the effect of the pro-inflammatory cytokine TNF-α on the tight junction (TJ) integrity and regulation of monolayer permeability in this BBB *in vitro* model (Fig. 3 a and see also Methods). TNF-α is a prominent cytokine involved in neuroinflammatory conditions and influencing BBB properties, and therefore was used here to examine whether it alters the interaction of exosomes with BMECs. As TNF-α is known to induce activation of nuclear factor kappa B (NF-κB) cascade, ultimately leading to apoptosis of some of the cells^70^, the potential induction of apoptosis in BMECs by TNF-α was monitored using XTT proliferation assay. XTT assays revealed that intermediate concentrations of TNF-α did not significantly alter cell viability and therefore a concentration of 50 ng/mL was used throughout subsequent studies (Fig. S3). The permeability across native BMECs monolayer (measured by FITC-dextran crossing through the transwells) steadily decreased during 48 hours in culture (*P* < 0.0001, Fig. 3b) as the cells grew next to each other and established their typical junctions in a honeycomb pattern, as expected (Fig. 3c). In accordance with previous studies on how BMEC monolayer is affected by TNF-α ^7, 47, 57^, the permeability of the BMEC monolayer that was treated by TNF-α was significantly increased compared to the control BMEC monolayer (*P* < 0.01, Fig. 3b), suggesting that activation by this cytokine compromised BMEC integrity.

Inflammatory processes have been reported to induce changes in F-actin and vascular endothelial cadherin (VE-cadherin) in endothelial cells concomitant with rearrangement of tight junction components^7^. As AJs and TJs play a major role in preventing passage through the BBB and maintaining intercellular tight junctions, we next examined if TNF-α alters the function of intercellular tight junctions. The VE-cadherin, zonula occludin-1 (ZO-1) and Claudin-5 expression (Fig. 3 c) were compared in cytokine activated and native conditions of BMECs. Concurrent with the increased paracellular permeability, treatment of confluent BMECs with TNF-α altered the expression of VE-cadherin, ZO-1 and Claudin-5, in which protein expressions were significantly decreased, suggesting a correlation in the levels and localization of these proteins with the integrity of AJs and TJs, as expected. In addition, TJ proteins (e.g., ZO-1) have been reported to shift from the membrane to cytoplasm and nuclei in ischemic hypoxia condition^15^. Similar results were also observed in our study in which immunostaining of TJ proteins showed a diffuse pattern at cell membrane, and some of them localized in the cytoplasm and nuclei (Fig. 3 c). Together, these data imply that TNF-α activation leads to a relocalization of AJ and TJ proteins and this disassembly (i.e., protein downregulation and their disengagement of cognate paracellular ligands) is likely responsible for the altered intercellular permeability of BMECs under inflammatory conditions.

### Exosomes cross TNF-α activated BMEC monolayer in vitro via transcellular route

**Fig. 4:**
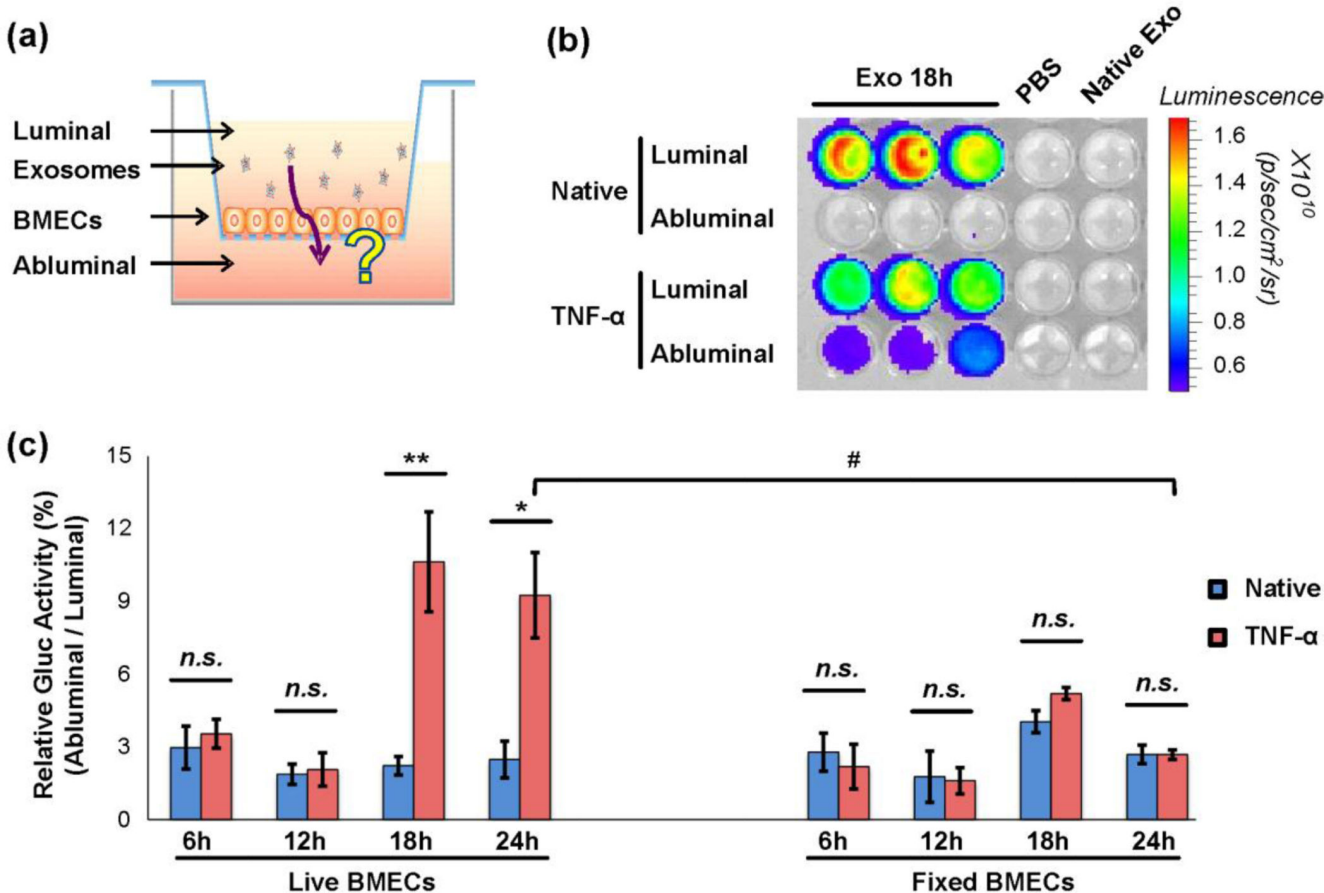
Exosomes can cross BMEC monolayer under stroke-like conditions in a transwell assay (a) Schematic representation of the *in vitro* model of the BBB. hGluc-Lact exosomes were added to the luminal chamber of the transwell and incubated with BMECs for various time points. Both luminal and abluminal chambers of conditioned medium were collected for bioluminescence assay. (b) and (c) Exosomes can cross BMECs in stroke-like conditions. (b) Representation of *in vitro* bioluminescence assay. Conditioned medium from both luminal and abluminal chambers were collected after exosome incubation and CTZ was added at a final concentration of 25 µM. *Gaussia* luciferase activity was measured immediately thereafter using IVIS Lumina (exposure time 0.5s). (c) Quantitative analysis of *in vitro* bioluminescence assay of hGluc-Lact exosomes crossing both live and fixed BMECs at different time points. Relative Gluc Activity = (abluminal chamber signal - native exo signal) / (luminal chamber signal -native exo signal) × 100%. Error bar: mean ± SEM. Native vs. TNF-α: *n.s*., not significant, *P < 0.05 and **P < 0.01. Live BMECs (TNF-α) vs. fixed BMECs (TNF-α) at 24 hours: #*P* < 0.05.

To investigate whether exosomes can cross the *in vitro* BBB model under normal and stroke-like conditions, transwell assays were performed using hGluc-Lact-labeled exosomes. Exosomes were added to the luminal chamber containing a confluent layer of BMECs (Fig. 4a), and conditioned medium from both the luminal and abluminal chambers were collected at several time points from 6 hours to 24 hours. hGluc activity in the collected conditioned medium was measured immediately after addition of the CTZ substrate. In transwell assays with native (untreated) BMECs, exosomes did not significantly cross the BMEC monolayer into the abluminal chamber, as shown by the lack of hGluc signal from the abluminal chamber and high signal from the luminal chamber (Fig. 4b and 4c). However, when BMECs were activated by TNF-α induction, significantly higher hGluc signals could be observed in the abluminal chamber, i.e. up to approximately 10% of exosomes crossed from the luminal to abluminal chamber after 18 hours (Fig. 4b and 4c). PBS and exosomes without hGluc used as negative controls did not reveal any significant bioluminescence, as expected (Fig. 4b). Thus, exosomes were able to cross the BMEC monolayer to the abluminal chamber, but only under the strokelike conditions with TNF-α activated BMECs. Furthermore, such exosome crossing was observed to be significantly increased for TNF-α activated BMECs after a minimum of 18 hours of incubation (*P* < 0.01, Fig. 4c).

**Fig. 5:**
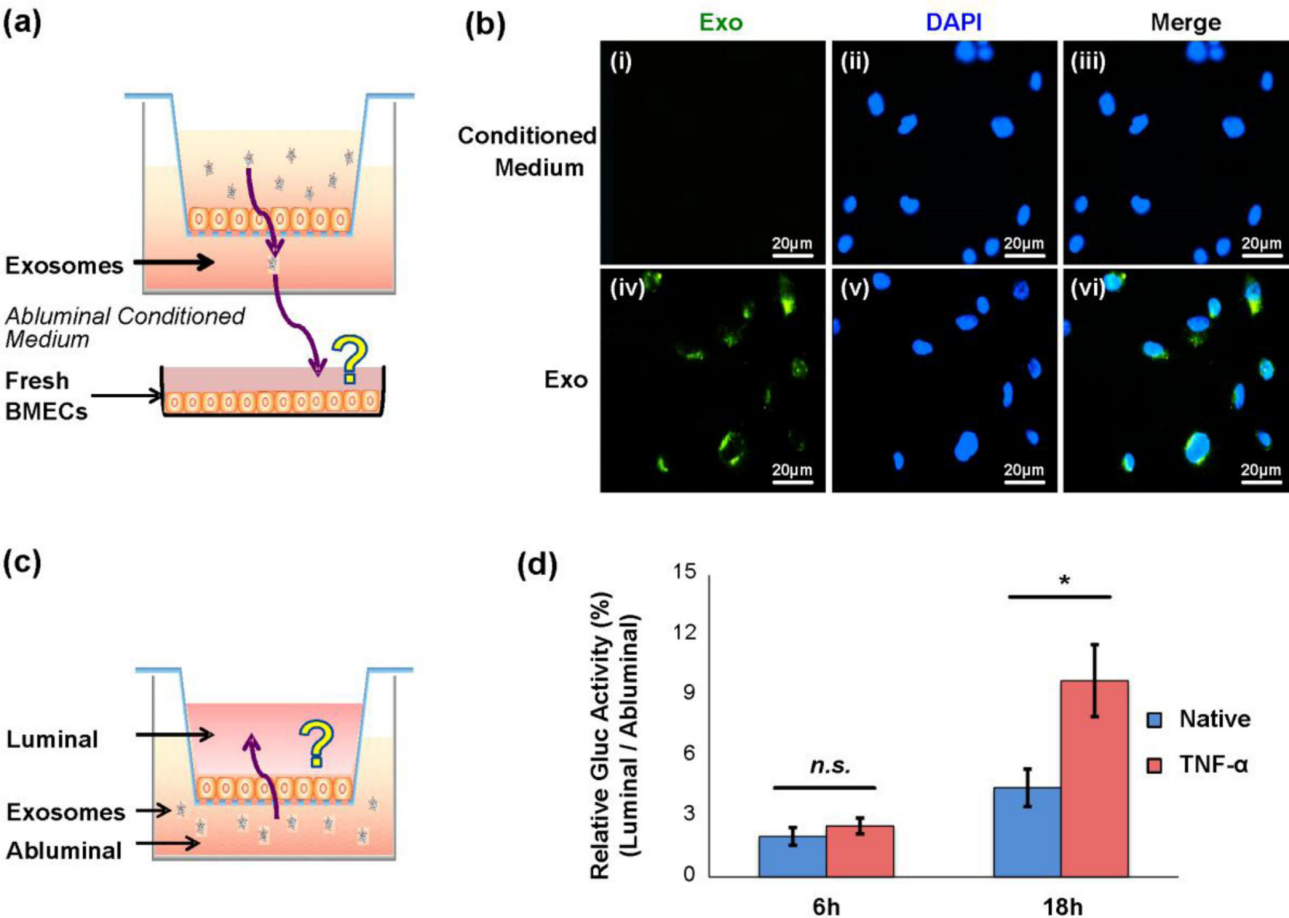
Validation of exosome crossing BMEC monolayers (a) Exosomes can cross the BMECs carrying hGluc *in vitro*. hGluc-Lact exosomes were labeled with the lipophilic dye PKH67, and were added to the luminal chamber of the transwell. Conditioned medium from abluminal chambers were collected and then incubated with a monolayer of BMEC on coverglass to further confirm the hGluc activity observed from bioluminescence assay was directly from exosomes. (b) Exosomes uptake by BMECs. hGluc conditioned medium of abluminal chamber stained with PKH67 was used as a control. Scale bar: 20 µm. (c) The schematic representation of exosome migration from abluminal chamber to the luminal chamber under native and TNF-α-treated conditions. (d) Quantitative analysis of exosome migration from abluminal to luminal chamber at 6 hours and 18 hours. Relative bioluminescence activity suggested that there was no significant difference between native and TNF-α-treated conditions at 6 hours, whereas the relative bioluminescence activity is significant higher in BMECs treated with TNF-α at 18 hours. Relative Gluc Activity = (luminal chamber signal - native exo signal) / (abluminal chamber signal - native exo signal) × 100%. Error bar: mean ± SEM. *n.s*., not significant and *P < 0.05.

As hGluc is a secreted protein with the molecular size 19kDa, it is expected to diffuse passively between the two chambers. Indeed, incubation of hGluc conditioned medium with BMECs in both native and TNF-α treated conditions revealed that hGluc can freely cross from luminal chamber to abluminal chamber in both conditions (Fig. S4). Therefore, to confirm that the hGluc activity observed from the transwell assays came directly from the exosomes instead of free hGluc detaching off the membrane, hGluc-Lact exosomes were directly labeled with PKH67 and added to the luminal chamber of a transwell assay with TNF-α activated BMECs. Conditioned medium from abluminal chamber was collected after 18 hours as above mentioned, and then added to a plate of fresh BMECs (Fig. 5a). PKH67 signal from exosomes can be seen clearly at perinuclear regions after 6 hours of incubation with fresh BMECs, (Fig. 5b, panel iv-vi), but conditioned medium containing free hGluc labeled with PKH67 used as a control displayed no signal, as was also the case abluminal chamber conditioned medium and fresh cells, as expected (Fig. 5b, panel i-iii). This, combined with the previous assay, demonstrated that exosomes can carry at least one defined protein cargo, in this case hGluc reporter enzyme as a model system, across a BMEC monolayer under stroke-like conditions, and therefore the hGluc activity observed from the transwell system was truly from the hGluc-exosomes instead of free hGluc.

In order to test if the difference in density between exosomes and growth medium has any effects on the BMEC monolayer crossing through gravity, hGluc-Lact exosomes were added to the abluminal (lower) chamber after BMEC stimulation with TNF-α (Fig. 5c). After 18 hours of incubation, hGluc activity was measured in the conditioned medium from the luminal (upper) chamber and was found to be significantly higher in BMECs activated with TNF-α compared to untreated (native) BMECs (*P* < 0.05, Fig. 5d). In other words, regardless of whether exosomes were added to upper or lower chambers, about 10% of them crossed the TNF-α treated BMEC monolayer, but they did not cross the untreated BMEC monolayer. Thus, the difference in exosome density and pull of gravity has little effects on their crossing, and it is equivalent to add exosomes to either upper or lower transwell chambers to study their crossing BMEC monolayers. Collectively, these results further suggest that exosomes can cross the activated, but not native, BMEC monolayer mimicking stroke-like conditions.

To further delineate by which mechanisms exosomes cross a BMECs monolayer, we next evaluated whether or not exosomes can also cross the *in vitro* BBB through passive diffusion of the paracellular route (i.e. though intercellular junctions between BMECs). In addition to a live BMECs transwell bioluminescence assay as in previous experiments, confluent BMEC monolayers (untreated or TNF-α activated) grown on the transwell insert were fixed with PFA, and exosomes were added for various time periods (6, 12, 18 and 24 hours) as before. Notably, no significant differences in bioluminescence activity were observed for fixed BMECs under both TNF-α activated and untreated conditions at all time points, unlike the much greater exosome crossing of the living BMECs under TNF-α compared to untreated condition. This data indicates that the paracellular diffusion route contributes little to the overall exosome crossing, i.e. no more than the background signal (e.g. native BMECs at 6 hours, Fig. 4c). Additionally, exosomes added to living BMECs treated with TNF-α showed significantly increased migration across the BMECs monolayer compared to that of exosomes added to fixed BMECs treated with TNF-α (for the 24 hour time point, *P* < 0.05, Fig. 4c). This suggested that the transcellular route, which operates only in the case of live but not fixed BMECs, likely accounts for majority of migration of exosomes across the BMEC monolayer.

### Exosomes are internalized by BMECs via endocytosis

**Fig. 6:**
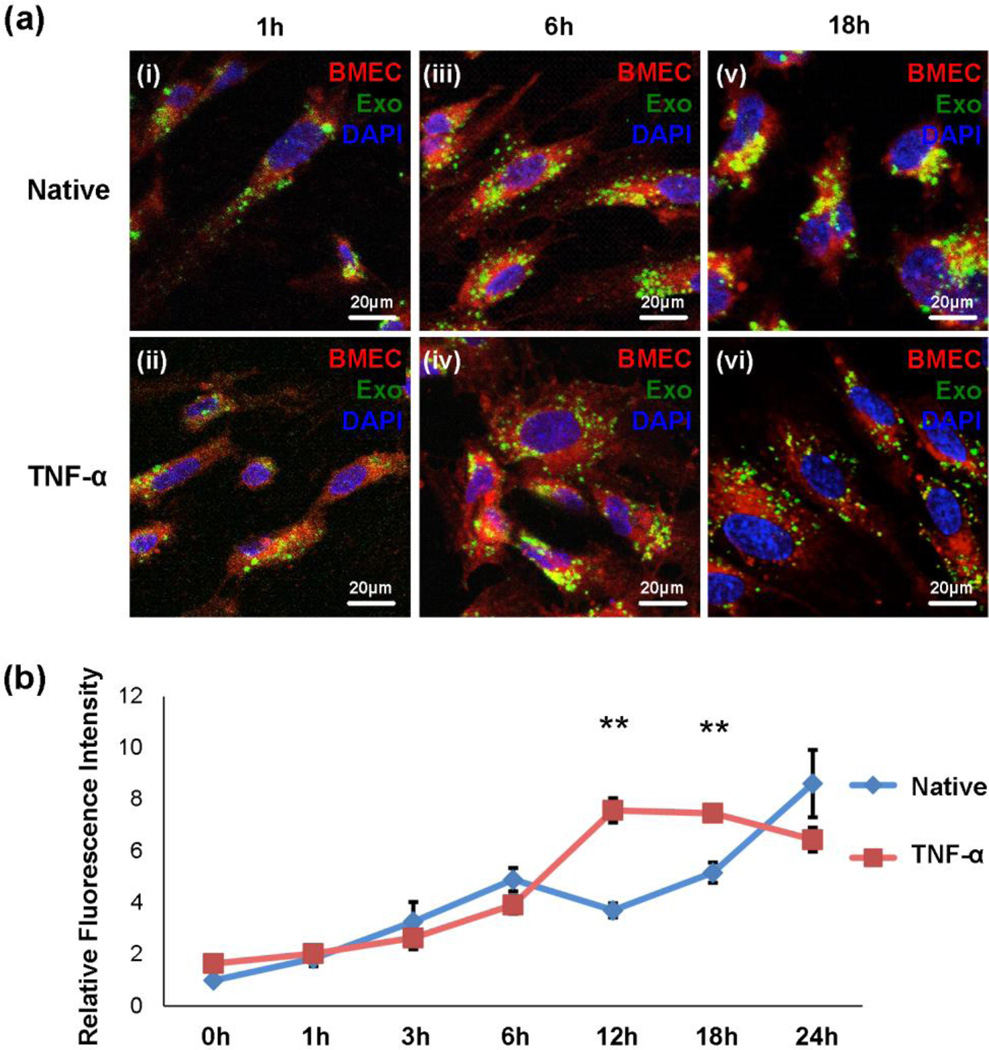
Exosome uptake by BMECs (a) and (b) Confocal microscopy analysis of exosome uptake by BMECs. (a) Representative pictures of exosome uptake under normal and stroke-like conditions at selected time points (1, 6 and 18 hour). Exosomes were labeled with PKH67 (green), BMECs were stained with CellMask (deep red), and DAPI (blue) was used for staining nuclei. Scale bar: 20 µm. (b) Quantitative analysis of fluorescence intensity of the PKH67-labeled exosomes. Briefly, the outline of each cell (*n* > 30) was drawn referring to the cell membrane labeling. The fluorescence intensity of intracellular exosomes that were specifically associated with the cells was then quantified (see Methods). Error bar: mean ± SEM. **P < 0.01.

Internalization of exosomes, MVB formation and subsequent exocytosis (i.e. transcytosis) is one proposed mechanism^55^ of exosome trafficking across the BMEC monolayer. To elucidate the mechanisms of exosome crossing in our study, microscopy experiments were performed to examine the interaction of exosomes with BMECs under both native and TNF-α activated conditions, and in the absence or presence of various endocytosis inhibitors. PKH67-labeled exosomes were incubated with CellMask-labeled BMECs (deep red) for 1, 3, 6, 12, 18 or 24 hours on coverglass. Confocal microscopy analysis was conducted to measure exosome uptake by BMECs (Fig. 6a and 6b). Briefly, cells were washed to remove any membrane-bound exosomes before sample preparation and the fluorescence intensity of intracellular exosomes that were specifically associated with the cells was then quantified (Fig. 6b, see also Methods). Fluorescence intensity analysis demonstrated that BMECs uptake exosomes in a time dependent manner, as exosome internalization was increased with longer incubation time (Fig. 6b). Furthermore, cells treated with TNF-α showed more uptake than native cells at 12 and 18 hours (*P* < 0.05, Fig. 6b), consistent with previous studies in which TNF-α activation resulted in an increase in nanocarrier internalization in brain endothelial cells^19^. Therefore, these data indicated that exosomes can be robustly internalized by BMECs. Native and TNF-α activated BMECs were observed to endocytose exosomes at similar rates up to 12 hours. Then the endocytosis of native cells kept increasing (*P* < 0.05, Fig. 6b) while that of TNF-α activated BMECs remained the same (*P* = 0.15, Fig. 6b). Interestingly, discrepancy between uptake (Fig. 6) and transwell (Fig. 4) assays was observed in that 1) the transwell data showed no significant exosome bypassing BMECs, regardless native and TNF-α activated conditions, within 12 hours, while uptake data demonstrated exosomes are being internalized by both native and TNF-α activated BMECs within the same time period, and 2) exosomes only bypass TNF-α activated but not native BMECs (at later time points (i.e., 18 h and 24 h)), whereas exosomes can be uptaken by both native and TNF-α activated BMECs. The reason for these observations remains unclear, however, it could be due to the active and unregulated exocytosis processes in TNF-α activated BMECs.

**Fig. 7:**
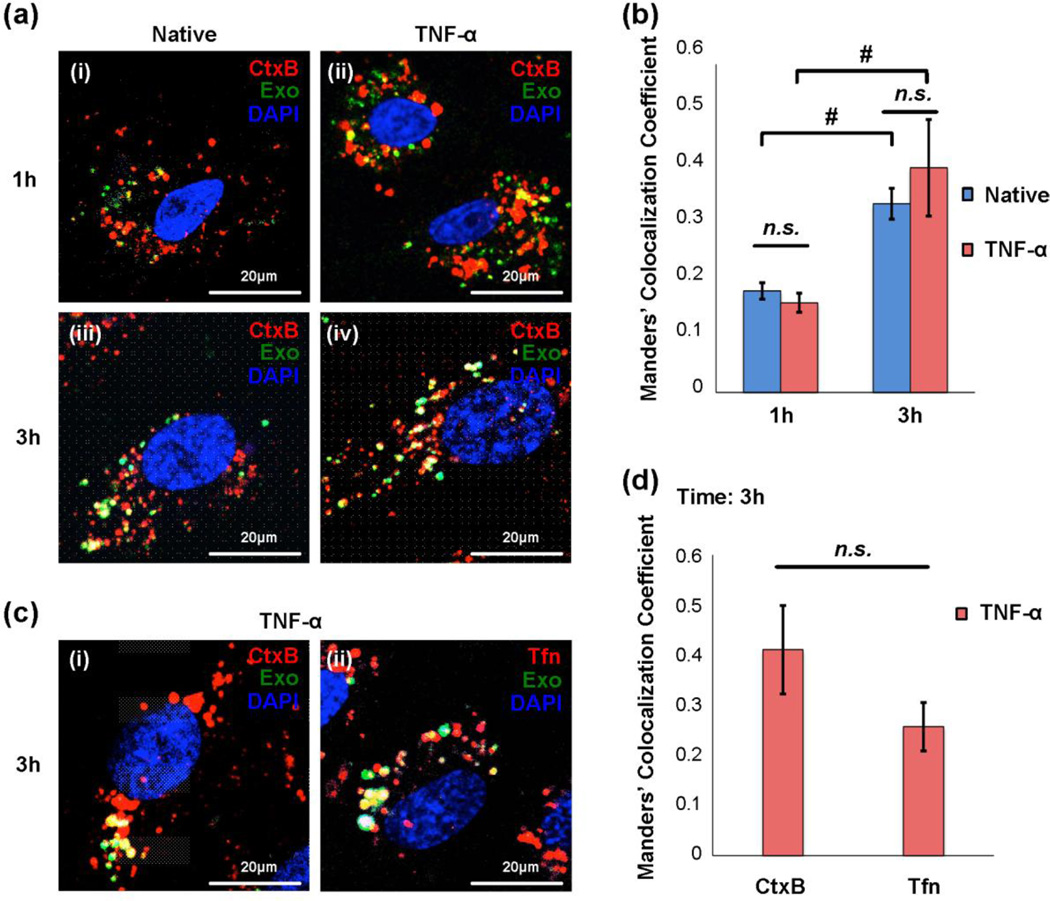
Exosome colocalization with early and late endosomes (a and b) Confocal microscopy analysis shows colocalization of cholera toxin B (CtxB) with exosomes. (a) Representative pictures of colocalization of CtxB with exosomes under normal and stroke-like conditions at 1 and 3 hours. Endosomes of BMECs (red, native or TNF-α stimulated) were stained with CtxB-biotin conjugated with Alexa Fluor 594-streptavidin for 30 minutes and then washed. PKH67-labeled exosomes (green) were incubated with BMECs for 1 or 3 hours. DAPI was used for staining nuclei. Scale bar: 20 µm. (b) Quantitative analysis of colocalization of CtxB with exosomes. Manders’ Colocalization Coefficient denotes the fraction of endosome membrane co-localized with exosomes. The coefficient tends to “1” if the exosomes are highly colocalized with endosomes. Error bar: mean ± SEM. Native vs. TNF-α: *P < 0.05. Native 1 hour vs. 3 hours or TNF-α 1 hour vs. 3 hours: ^#^*P* < 0.05. (c and d) The colocalization of early (Tfn) and late (CtxB) endosomes and exosomes. (c) Representative pictures of colocalization of (i) CtxB and (ii) Tfn with exosomes under stroke-like conditions at 3 hours. PKH67-labeled exosomes (green) were incubated with TNF-α stimulated BMECs for 3 hours, and then endosomes of BMECs (red) were stained with CtxB-Alexa Fluor 594 or Tfn-Texas Red; DAPI was used for staining nuclei. Scale bar: 20 µm. (d) Quantitative analysis of (c) to measure the association of exosomes with CtxB or Tfn. Error bar: mean ± SEM. *n.s*., not significant.

In order to study the intracellular trafficking of internalized exosomes in BMECs, early and late endosomal markers were used to identify endosomal trafficking machinery. BMECs were pre-incubated with cholera toxin B (CtxB), a late endosomal compartment marker, and cultured with PKH67-labeled exosomes for 1 or 3 hours. Confocal microscopy analysis revealed that colocalization of exosomes and CtxB was observed at 1 hour (Fig. 7a, panel i and ii), and accumulation of colocalization was significantly increased at 3 hours in both native and TNF-α treated BMECs (*P* < 0.05, Fig. 7a, panel iii and iv and 7b), in agreement with our previous data showing the uptake of exosomes in a time-dependent manner (Fig 6a and 6b). Internalized exosomes were also observed to co-localize with transferrin (Tfn)-Texas Red, an early endosome marker, in BMECs (Fig. 7c and 7d). Taken together, these data suggest that exosomes can be internalized by BMECs and subsequently trafficked via endocytic mechanisms.

**Fig. 8:**
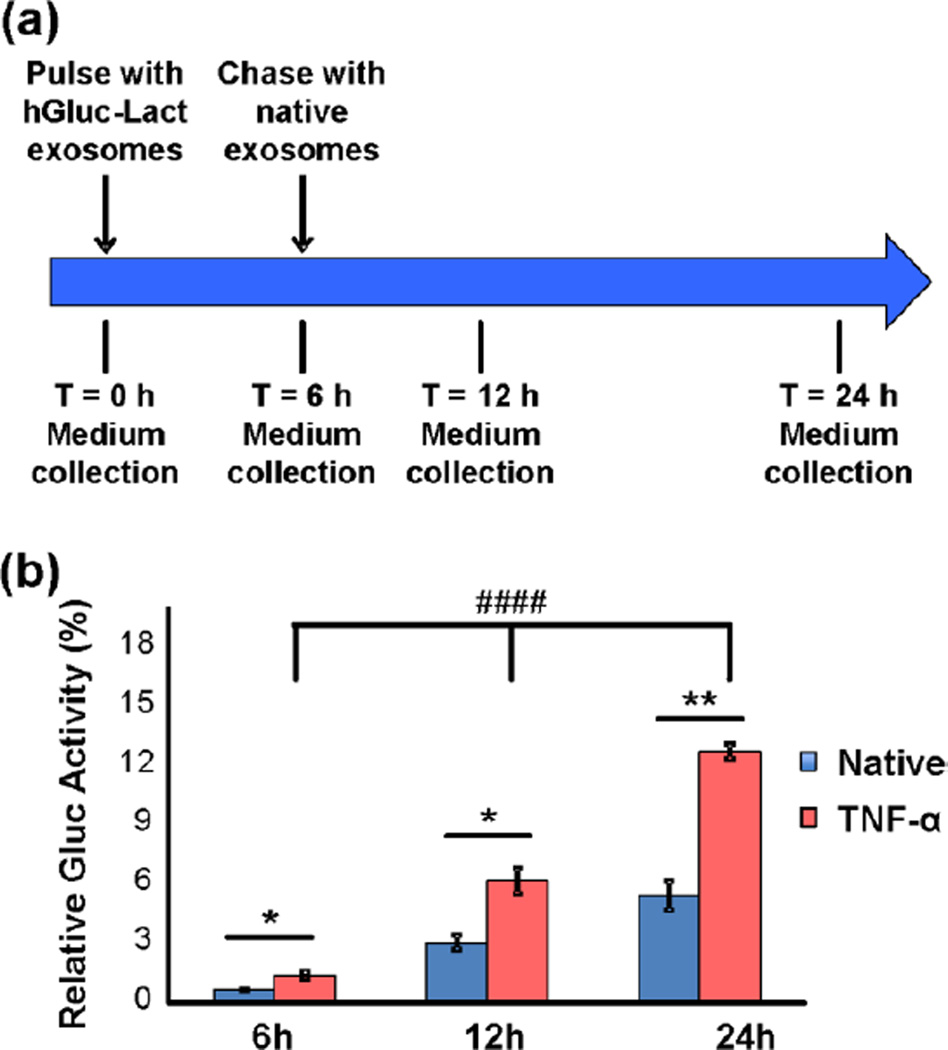
Exosomes were exocytosed by BMECs in a transwell assay (a) Illustration of the pulse-chase experiment to study exosome exocytosis *in vitro*. (b) Conditioned medium from BMECs pulsed for 6 hours with hGluc-labeled exosomes and chased with unlabeled exosomes (at indicated time points) was collected from both luminal and abluminal transwell chambers at indicated time points and hGluc activity was measured using IVIS Lumina (CTZ final concentration: 25 µM, exposure time: 0.5s). Error bar: mean ± SEM. Native vs. TNF-α: *P < 0.05 and **P < 0.01. Under TNF-α condition: ^####^*P* < 0.0001, as determined by one-way ANOVA with SNK post-hoc test.

To study the active transcellular route of exosomes across the BMEC monolayer *in vitro*, a pulse-chase analysis and transwell assay were employed. Pulse-hGluc-Lact exosomes were added to the luminal chamber and incubated with cells for 6 hours. For exosome chasing, hGluc-Lact exosomes were then removed and cells were washed prior to addition of unlabeled exosomes. Cells were then incubated with unlabeled exosomes for 6 and 18 hours (Fig. 8a). Conditioned media from both luminal and abluminal chambers were collected, and luciferase activity was measured from combined luminal and abluminal chambers (since BMECs are expected to secrete on both sides) and normalized to total hGluc-Lact exosomes at 0 h. In other words, the relative luciferase activity of secreted exosomes over total input exosomes indicated the percentage of exosomes secreted by BMECs. Bioluminescence activity was observed in both luminal and abluminal chambers, revealing the secretion of hGluc-Lact exosomes by exosome-pulsed BMECs in a time dependent manner (*P* < 0.0001 Fig. 8b). Importantly, a significant increase of exosome secretion was found in the TNF-α stimulated condition compared to unstimulated condition (*P* < 0.05, T = 6 h; *P* < 0.05, T = 12 h and *P* < 0.01, T = 24 h, Fig. 8b), which is consistent with the observation of increased exosome uptake by BMECs leading to subsequent increased exocytosis.

### Exosome internalization by BMECs includes clathrin-dependent and caveolae-dependent routes

**Fig. 9:**
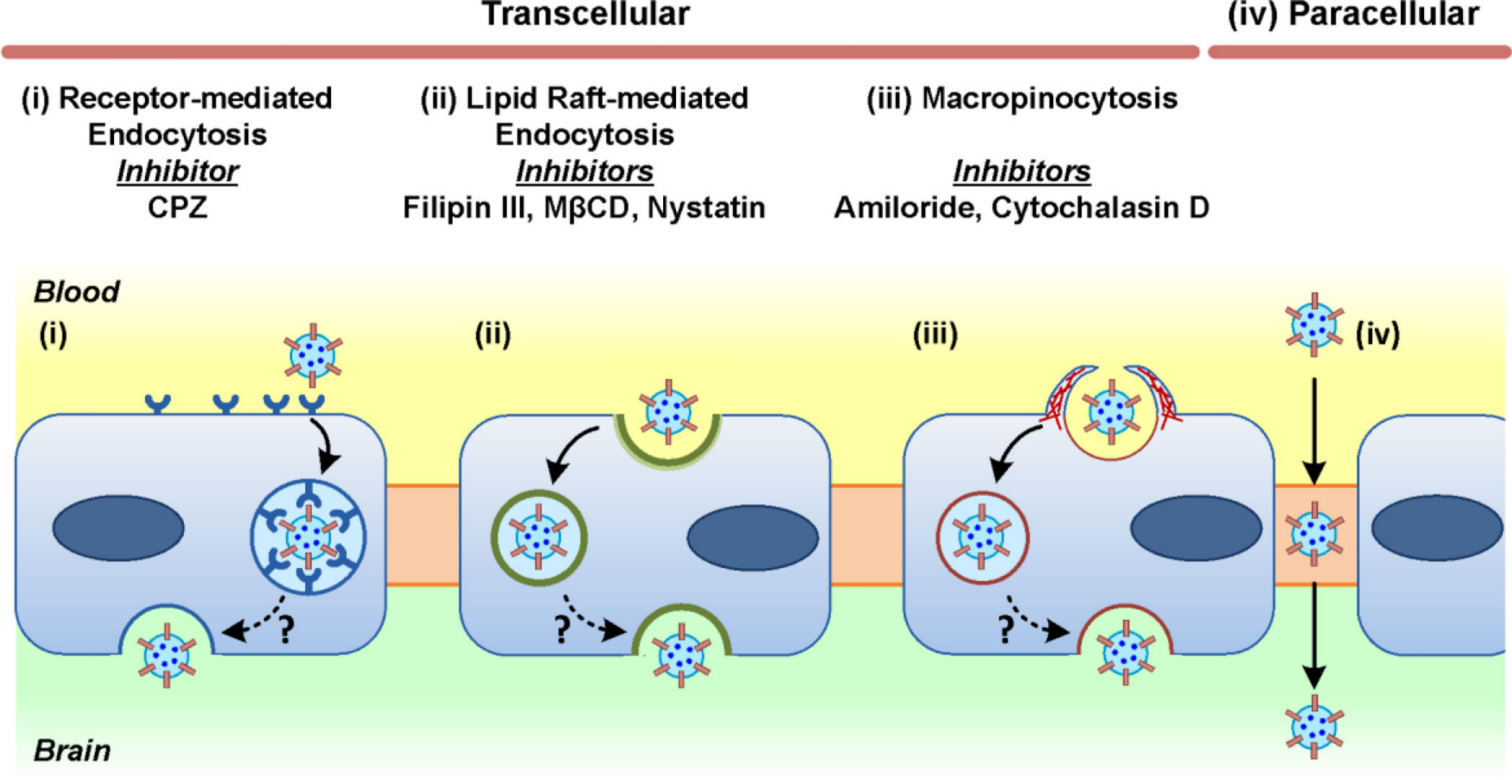
Potential mechanisms of exosome crossing the blood-brain barrier (BBB) Schematic representation of the proposed mechanisms on how exosomes cross the BBB, and diagram of the major endocytic pathways as well as their corresponding inhibitors used in this study.

**Fig. 10:**
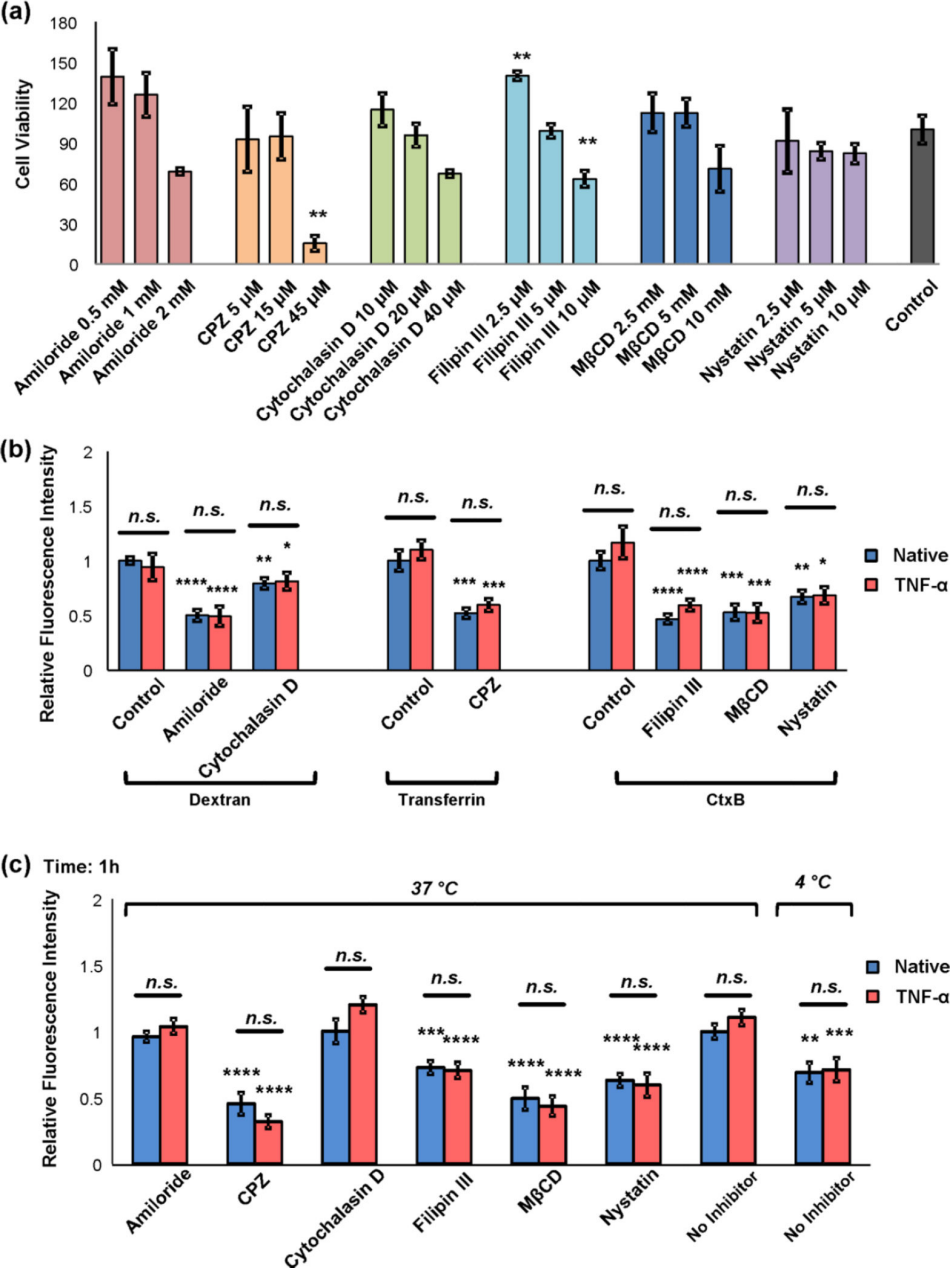
Effects of endocytosis inhibitors on exosome internalization (a) BMECs were treated with the indicated endocytosis inhibitors for 30 minutes, and then cell viability was determined by XTT assay. Relative cell viability was normalized to the vehicle control (no inhibitor) set as 1. Error bar: mean ± SEM. *P < 0.05 and **P < 0.01, as determined by one-way ANOVA with SNK post-hoc test. (b) BMECs were pretreated with inhibitors or vehicle (no inhibitor), followed by incubation with Texas Red-transferrin, Alexa Fluor 594-CtxB, or Alexa Fluor 594-dextran. Cellular uptake was measured by fluorescence microscopy and fluorescence intensity was quantified and displayed in the bar graphs. Error bar: mean ± SEM. *n.s*., not significant, *P < 0.05, * *P < 0.01, ***P < 0.001 and ****P < 0.0001. (c) BMECs were pretreated with indicated inhibitors: amiloride (1mM), CPZ (15 µM), cytochalasin D (20 µM), filipin III (5 µM), MβCD (5 mM), nystatin (5 µM) for 30 minutes at 37°C. Exosomes labeled with PKH67 were incubated with BMECs at 37°C for 1 hour, and their uptake was imaged with confocal microscopy and quantified as described in Methods. Error bar: mean ± SEM. Native vs. TNF-α: *n.s*., not significant, **P < 0.01 and ****P < 0.0001. Conditions compared were: native (unstimulated BMECs) in the absence of inhibitors vs. in the presence of inhibitors, and native BMECs vs TNF-α-stimulated BMECs in the absence or presence of inhibitors: *P < 0.05, **P < 0.01, ***P < 0.001 and ****P < 0.0001, compared to native no inhibitor or TNF-α no inhibitor conditions, respectively.

As previous studies have reported that cell uptake of exosomes includes an active endocytosis mechanism^55, 34^, using uptake assay we next examined whether or not exosome uptake by BMECs and transmigrate the BMEC monolayer is an energy-requiring process or a passive membrane diffusion process (Fig. 9). Incubation of cells with exosomes at 4°C for 1 hour significantly reduced exosome uptake compared to the usual 37 °C incubation, indicating the operation of an energy-dependent process (*P* < 0.05, Fig. 10c). Exosomes can be internalized into target cells through different mechanisms, including phagocytosis, macropinocytosis, clathrin-mediated endocytosis (CME), lipid raft (caveolae)-mediated endocytosis and plasma membrane fusion^34, 55, 60^. Therefore, we further investigated which endocytic pathways were most involved in exosome entry into BMECs. Various pharmacological inhibitors of endocytic transport, including amiloride, a Na^+^/H^+^ exchanger to block macropinocytosis; filipin III, methyl-β-cyclodextrin (MβCD), nystatin, to inhibit lipid raft/caveolae-mediated endocytosis; chlorpromazine (CPZ), to inhibit clathrin-dependent endocytosis, and cytochalasin D, to depolymerize actin and inhibit fluid-phase macropinocytois were used to examine exosome transcellular trafficking across the BMECs monolayer (Fig. 9).

As cytotoxicity and specificity of endocytosis inhibitor were reported to be cell-type dependent^69^, the cellular viability of BMECs after treatment with the various concentrations of endocytosis inhibitors was measured using XTT assay. Cellular viability was significantly reduced only at high concentration of most inhibitors, and therefore the concentrations of endocytosis inhibitors used in all subsequent studies were: CPZ (15 µM), cytochalasin D (20 µM), amiloride (1mM), methyl- β -cyclodextrin (MβCD, 5 mM), filipin III (5 µM), or nystatin (5 µM) (Fig. 10a). Next, we examined the effectiveness and specificity of endocytosis inhibitors at the concentrations determined above using endocytic markers specific for each endocytosis mechanism. Transferrin, CtxB, and dextran were shown to be internalized by CDE, caveolae-mediated endocytosis, and macropinocytosis, respectively^55, 63^. Treatment with amiloride and cytochalasin D were shown to specifically inhibit macropinocytosis, and CPZ was revealed to significantly inhibit CDE. Treatment with filipin III, MβCD, or nystatin prior to CtxB incubation exhibited a significant reduction in dye uptake, confirming a blockage of caveolae-mediated endocytosis (Fig. 10b).

**Fig. 11:**
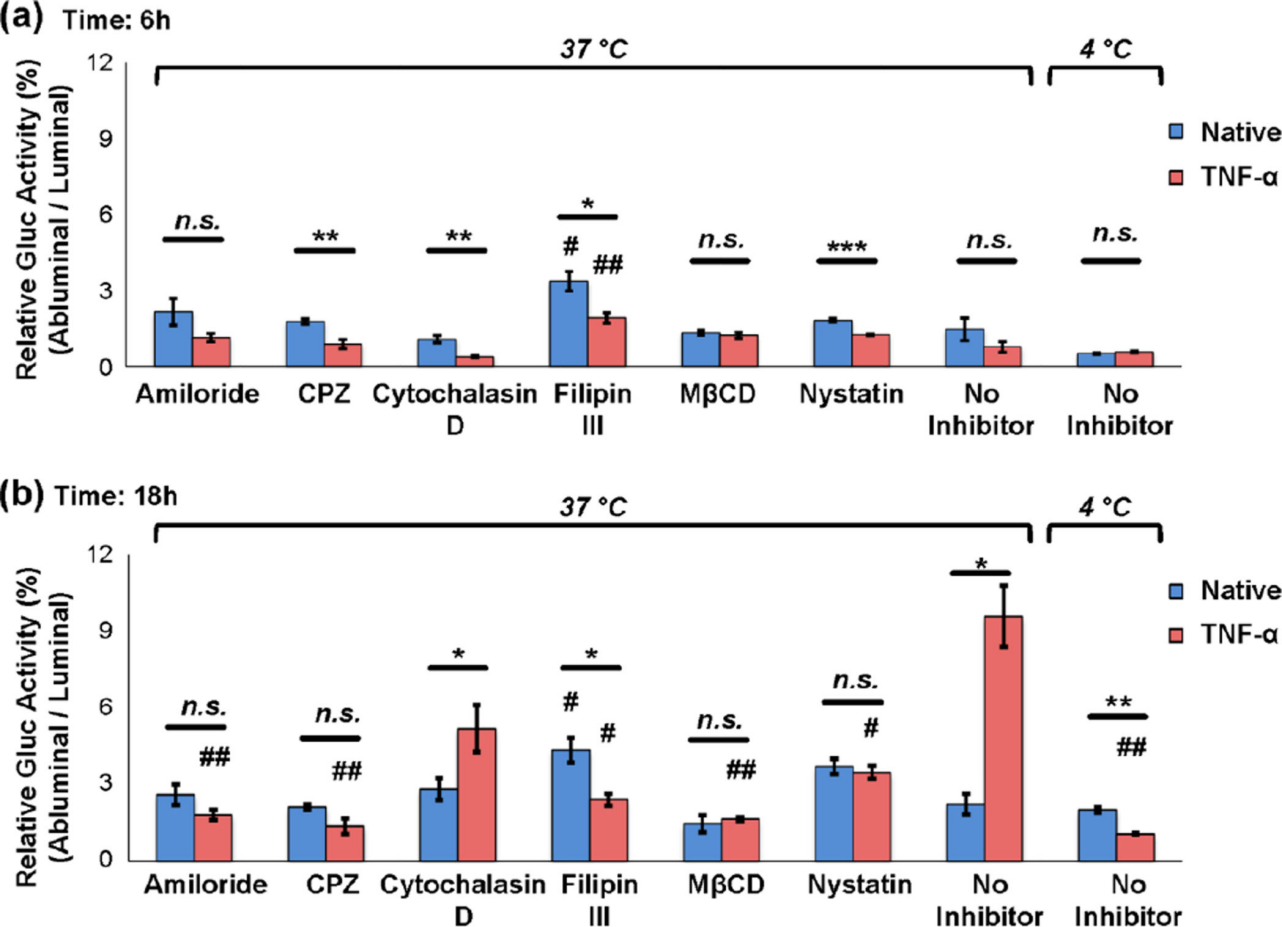
Inhibition of endocytosis decreases exosome crossing the *in vitro* BBB BMECs were pretreated with indicated inhibitors: amiloride (1mM), CPZ (15 µM), cytochalasin D (20 µM), filipin III (5 µM), MβCD (5 mM), nystatin (5 µM) for 30 minutes at 37°C, respectively. hGluc-Lact exosomes were subsequently added to the luminal chamber of each transwell and incubated with BMECs for various time points (6 and 18 hours). Cells treated with vehicles (no inhibitor) alone were used as a negative control. To study the temperature effect on endocytosis, BMECs containing exosomes were incubated at either 37°C or 4°C for (a) 6 and (b) 18 hours, and then conditioned medium from both luminal and abluminal chambers were collected and *Gaussia* luciferase activity was measured immediately after addition of its substrate CTZ (IVIS Lumina, exposure time: 0.5s). Relative Gluc Activity = (abluminal chamber signal -native exo signal) / (luminal chamber signal - native exo signal) × 100%. Error bar: mean ± SEM. Native vs. TNF-α: *n.s*., not significant, *P < 0.05 and **P < 0.01. #*P* < 0.05 and ##*P* < 0.01, compared to native no inhibitor or TNF-α no inhibitor conditions, respectively.

To study which mechanisms were mostly involved in exosome endocytosis pathways, cells were pretreated with endocytosis inhibitors for 30 minutes follow by exosomes incubation for 1 hour. Exosome uptake was significantly inhibited by filipin III (73% for native, *P* < 0.001 and 64% for TNF-α, *P* < 0.0001, respectively), MβCD (49% for native, *P* < 0.0001, and 39% for TNF-α, *P* < 0.0001, respectively), and nystatin (63% for native, *P* < 0.0001, and 54% for TNF-α, *P* < 0.0001, respectively) (Fig. 10c), suggesting lipid raft disruption blocked exosome internalization. Therefore, caveolae-dependent endocytosis is one likely route of exosome internalization. Similarly, clathrin-dependent endocytosis inhibition by CPZ attenuated uptake of exosomes in BMECs (45% for native and 29% for TNF-α, *P* < 0.0001), also suggesting the involvement of this endocytic pathway. To further corroborate these data, the transwell bioluminescence assay was employed to estimate the contribution of the different endocytosis mechanisms under native and activated (TNF-α) conditions. Pretreatment of most inhibitors followed by 6 hours’ incubation of exosomes showed similar results of exosome crossing in both native and TNF-α treated BMECs which are minimal in both cases (Fig. 11a). Interestingly, filipin III showed a promotional effect on exosome migration in both conditions. This is probably due to a high concentration of filipin III (5 µM) used in this study, which led to membrane permeabilization and an increase of exosome transport^51^. Most inhibitor treatments resulted in a significant reduction of exosome migration from luminal to abluminal chamber at 18 hours most notably in the case of TNF-α treated BMEC system while minimal exosome bypassing native BMECs was observed with or without inhibitor treatment (Fig. 11b). The attenuation of exosome crossing by inhibition of endocytosis indicated that exosomes cross TNF-α treated BMEC monolayer largely via the transcellular route. Importantly, multiple endocytosis mechanisms (as judged by the effects on exosome crossing exerted by their corresponding inhibitors) are likely involved in exosome transcellular migration. For example, abrogation of caveolae-mediated endocytosis by cholesterol-depleting agents MβCD and filipin III resulted in significant reductions in exosome transcellular migration (Fig. 11). Likewise, inhibition of clathrin-dependent endocytosis by CPZ (which decreases the formation of clathrin-coated pits at the plasma membrane) suggested that the uptake and transport of exosomes across the BMECs is also dependent on clathrin-mediated endocytosis (Fig. 11). However, cytochalasin D, which has been reported to increase dextran uptake and decrease microparticle uptake in human brain endothelial cells^14^, here altered exosome intracellular trafficking in both native and TNF-α stimulated conditions. Moreover, another inhibitor, amiloride, which has been reported to alter intracellular pH homeostasis, here also reduced exosome migration during the TNF-α activated condition. One possible explanation could be that changes in intracellular pH delayed altered lysosome acidification and subsequently diminished exosome exocytosis.

Intriguingly, a relative reduction in exosome crossing through the BMEC monolayer was observed when cells were treated with TNF-α at 4°C compared to the native BMECs at 4°C (*P* < 0.01, Fig. 11), suggesting low temperature might also attenuate TNF-α induced changes in the BBB barrier function. Additionally, exosomes were incubated at 37°C and 4°C under the complete absence of cells to determine any measurable temperature effects on the exosome diffusion rate. No significant differences between 37°C and 4°C were observed when incubating exosomes for 6, 18, and 24 hours in the absence of a BMEC monolayer (Fig. S5), suggesting that temperature effects did not affect the background diffusion of exosomes.

These inhibitor studies are generally consistent with clathrin and caveolae-mediated mechanisms of exosome internalization of BMECs suggested by examination of the colocalization of early and late endocytic markers. That is, colocalization of exosomes and Tfn, which is a marker of clathrin-mediated endocytosis, confirmed the exosome uptake by BMECs involves clathrin-dependent endocytosis mechanism (Fig. 7c and 7d). Likewise, colocalization of exosomes and CtxB, which is a marker of caveolae-mediated endocytosis, verified that exosome internalization is associated with lipid raft-mediated endocytosis (Fig. 7a and 7b). Together, these data suggested that exosomes could cross the *in vitro* BBB model through primarily transcellular routes rather than the passive paracellular route. For the transcellular crossing, both clathrin-mediated endocytosis and caveolae-mediated endocytosis likely play important roles in exosome transport.

## CONCLUSIONS

The BBB, also referred to as the blood-brain interface^4^, protects the brain from harmful chemicals or toxins from the systemic circulation, yet it also results in the inability of most therapeutics to cross the BBB and reach the CNS. So far, several approaches have been investigated to increase the transport of therapeutics to the brain, including local invasive intracerebroventricular (ICV) infusion, intranasal administration, induction of permeability by temporary disruption of the BBB (e.g., by ultrasound), use of prodrugs that can bypass BBB, pharmacological strategies using colloidal drug carriers such as nanoparticles or liposomes, more complex methods using endogenous transport mechanisms (either receptor mediated transport of chimeric proteins or carrier mediated transport of nutrients), and transient inhibition of drug efflux mechanisms^4, 10, 13, 38, 39^. Nevertheless, delivering therapeutic and diagnostic agents across the BBB remains a daunting challenge.

Exosomes are involved in a variety of activities in the CNS, including neuron-glia communication, inducing neurite outgrowth and neuronal survival, transfer of toxic proteins including β-amyloid peptide that contributes to pathogenic amyloid-β deposition, mediation of neuronal development, and modulation of synaptic activity^12, 16, 21^. Endogenously as well as exogenously administered exosomes may be potential therapeutic agents to mediate and treat neurological diseases^74^. Moreover, under certain pathological conditions including stroke and traumatic brain injuries, exosomes were shown to have significant therapeutic effects and neurological improvements^74, 76^. In addition, extracellular vesicles (EVs) have been found to mediate, in part, the curative effects of cell-based therapies for CNS diseases^50, 73^. Thus, understanding the mechanisms of how EVs, particularly exosomes, may bypass the BBB to exert their curative effects or deliver intended cargo is a prerequisite to developing EVs as carriers of therapeutic or diagnostic agents for CNS diseases.

Here, we investigated the interactions between BMECs and exosomes trafficking *in vitro* under healthy and stroke-like conditions. Exosomes labeled with hGluc could readily be detected via bioluminescence, which allowed us to evaluate exosome trafficking quantitatively *in vitro* and will likely enable their use in future studies on EV biodistribution *in vivo* in both the healthy and pathological CNS. In particular, we observed that not only compromised TJs, but also are other cell functions including endocytosis were affected by cell activation with the inflammatory cytokine TNF-α (i.e., under stroke-like conditions)^9^. Specifically, we demonstrated that luciferase-carrying exosomes (hGluc-Lact exosomes) could be internalized by BMECs, and cross BMECs more effectively under stroke-like conditions *in vitro*. Using confocal microscopy, we demonstrated that exosomes are internalized by BMECs through endocytosis and accumulate in endosomes. In addition, the majority of exosome crossing the BMEC monolayer was via the transcellular route (i.e. endocytosis, MVB formation and exocytosis across the other side of the layer), with little via the paracellular route (i.e. via passive diffusion though intercellular gaps between BMECs). However, as a caveat of this study, the exosome exocytosis and intracellular fate of exosomes were not investigated in this study. It is likely that a fraction of the exosomes fuse with lysosomes resulting in their digestion. Also, *de novo* generated exosomes are expected to be secreted by BMECs, in part influenced by events triggered by internalization of exogenous exosomes. A major fate of internalized exosomes is through the formation of MVBs that rely on multi-subunit endosomal sorting complex required for transport (ESCRT) machinery^62^. Internalized exosomes as well as *de novo* generated exosomes are termed intraluminal vesicles (ILVs). MVBs hosting ILVs can traffic to and fused with the plasma membrane to liberate exosomes^37^, in the process utilizing Rab family and small GTPases. In addition, the endosome associated proteins including soluble N-ethylmaleimide-sensitive factor attachment protein receptor (SNARE) proteins and annexins have also been proposed to involve in mediating vesicular trafficking of MVBs and regulating exosome release from the plasma membrane^61, 6^. A different possible fate of internalized exosomes is the fusion of MVBs with lysosomes to degrade the exosomes/ILVs. Furthermore, different pathways of exosome secretion can differ between different cell types, with BMECs particularly active in coupled edocytosis-exocytosis to yield efficient transcytosis of molecules such as albumin, antibodies and various growth factors^22, 45^. The intracellular trafficking of exosome secretion by BMECs after endocytosis was not examined in detail in this present study, but future studies are expected to further elucidate the exocytic trafficking of exosomes in the context of healthy and pathological BBB conditions^62^.

Our data suggest that exosomes derived from 293T cells can be internalized by multiple pathways of endocytosis, including clathrin-dependent and caveolae-dependent routes. Nonetheless, since cellular uptake of exosomes depends on specific ligand receptors or lipid rafts^55^, future studies should focus on more precisely identifying the molecular mechanisms at play on the interaction of exosomes with BMECs. For example, by using engineered exosomes from different cellular sources that carry therapeutic potential, functional antibodies to block receptor-ligand interactions, or siRNA to knockdown specific genes that involves in endocytic process for exosome uptake, their MVB formation, their exocytosis as newly released exosomes and their passive diffusion across BMEC intercellular gaps may further unravel molecular mechanisms of exosomes migration across the BBB.

This study therefore paves the way for further development of engineered exosomes as drug delivery vehicles or tracking tools for treating or monitoring neurological diseases. Although *in vitro* BMEC monolayers recapitulate many of the characteristic features of the in *vivo* BBB, incorporation of other cell types, such as primary astrocytes, pericytes, and reconstituted basement membrane may provide a more robust model. As exosome composition varies from one cell type to the other^61^, the intercellular communication and cellular trafficking between exosomes and BMECs might depend on the cell source and their treatment prior to exosomes production. Although exosomes produced by the cell line HEK 293T used in this study have been engineered and used for potential drug delivery vesicles^36^, EVs derived from cell types that have been widely used in translational applications, including MSCs and immune cells^30^, are likely more ideal candidates to derive EVs for therapeutic use in clinic and will be considered in the future studies.

Moreover, additional *in vivo* and *ex vivo* assays are expected to further elucidate the physiological mechanisms of exosomes crossing the BBB and their biodistribution in the healthy and pathological CNS in animal models. While here we did not study exosomes biodistribution in the brain, it would be interesting to employ our sensitive hGluc exosomes labeling system in brain slices and in live animals *in vivo*. This system can be used to examine whether or not exosomes can be utilized to deliver therapeutic agents to specific regions of the injured brain. Taken together, our study provides insights into the development of exosomes as emerging therapeutic vehicles and diagnostic tools in the near future. The ease of bioengineering and superior biodistribution characteristics of exosomes may enable their likely therapeutic roles as drug delivery vehicles, in addition to their better-studied intrinsic curative abilities.

## Supplementary Material

12195_2016_458_MOESM1_ESM

12195_2016_458_MOESM2_ESM

12195_2016_458_MOESM3_ESM

12195_2016_458_MOESM4_ESM

12195_2016_458_MOESM5_ESM

12195_2016_458_MOESM6_ESM
