# Supplementary material for: Elucidation of Exosome Migration across the Blood-Brain Barrier Model In Vitro
Source: Cell Mol Bioeng. Author manuscript; Available in PMC 2017 Dec 1. (PMC5382965; doi:10.1007/s12195-016-0458-3)
Supplement: 12195_2016_458_MOESM1_ESM [file NIHMS801559-supplement-12195_2016_458_MOESM1_ESM.docx]

**Fig. S1** **Membrane fusion protein hGluc-Lact is expressed in 293T cells.**

(a) Schematic representation of the hGluc-Lactadherin constructs engineered in this study. (b) Western blot analysis showing 293T cells expressing membrane fusion proteins hGluc-Lact and hGluc-Lact-GFP. Membranes probed with anti-Gluc antibody showed *Gaussia* luciferase (Gluc) at the expected sizes (55 kDa, and 82 kDa, respectively) compared to hGluc alone (20 kDa). Membrane was probed with anti-GFP antibody and only GFP band was detected at 82 kDa in hGluc-Lact-GFP sample. β-actin was used as a loading control. (c) Quantification of hGluc expression of (b) showing relative expression of free hGluc and tagged hGluc in lysate. The band intensity was normalized to β-actin. Relative intensities were: hGluc = 0.0437, hGluc-Lact = 0.825 and hGluc-Lact-GFP = 0.727.

**Fig. S2** **Stability of hGluc-Lact exosomes under various temperature conditions.**

(a) Nanoparticle tracking analysis (NTA) of hGluc-Lact exosomes at 37°C, 25°C and 4°C. Incubation at different temperatures did not alter exosome size distribution. (b) *In vitro* bioluminescence assay of hGluc-Lact exosomes at different temperature conditions. Values are mean ± SEM of relative ratio normalized to 37°C set as 100%. There were no significant differences in luciferase activity between 37°C, 25°C and 4°C; *n.s.*, not significant.

**Fig. S3** **Effect of TNF-α on BMEC growth.** Cell viability was measured after 6 hours of TNF-α activation and determined by XTT assay. Relative cell growth was normalized to the vehicle control (no TNF-α) set as 100%. Error bar: mean ± SEM.**P* < 0.05 and ***P* < 0.01, as determined by one-way ANOVA with SNK post-hoc test.

**Fig. S4** **Diffusion of free hGluc from conditioned medium across BMEC monolayers.** Secreted hGluc protein from conditioned medium can freely cross BMECs in both native and activated conditions hGluc activity was measured using IVIS Lumina (CTZ final concentration: 25 μM, exposure time: 0.5s). Relative Gluc Activity = (abluminal chamber signal **–** hGluc free conditioned medium signal) / (luminal chamber signal **–** hGluc free conditioned medium signal) × 100%. Error bar: mean ± SEM. *n.s.*, not significant.

**Fig. S5** **Measurement of temperature effects on *in vitro* transwell diffusion of exosomes in the absence of cells (free diffusion)**

The free diffusion rate of exosomes in the absence of live or fixed BMECs was determined by transwell and *in vitro* bioluminescence assay. Conditioned media from both luminal and abluminal chambers were collected at 6, 18, and 24 hours after addition of exosomes to the luminal chamber. Incubation of exosomes at 37°C and 4°C did not alter the diffusion rates of exosomes. Error bar: mean ± SEM. *n.s.*, not significant.
