# Supplementary figures and images for: Elucidation of Exosome Migration across the Blood-Brain Barrier Model In Vitro
Source: Cell Mol Bioeng. Author manuscript; Available in PMC 2017 Dec 1. (PMC5382965; doi:10.1007/s12195-016-0458-3)

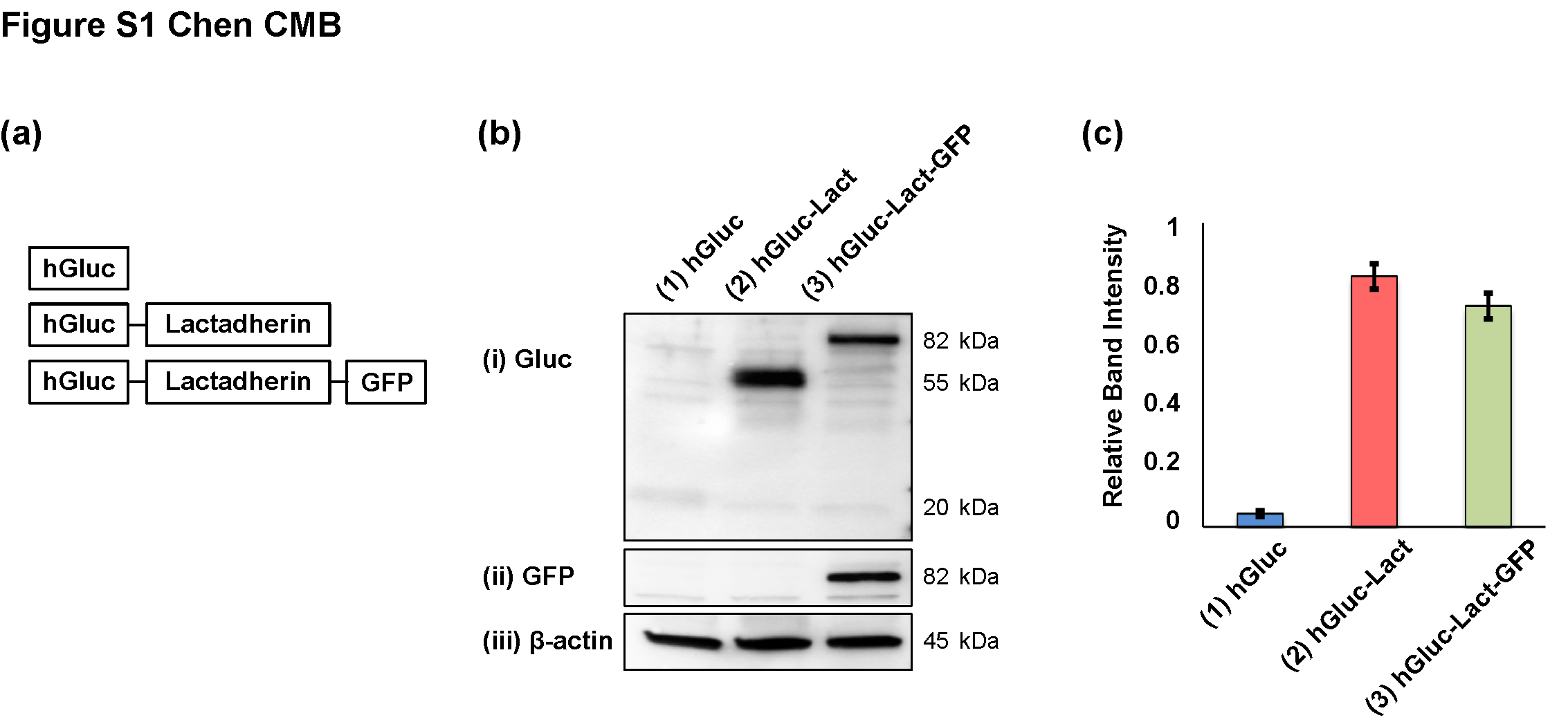

Supplement: 12195_2016_458_MOESM2_ESM [file NIHMS801559-supplement-12195_2016_458_MOESM2_ESM.tif]

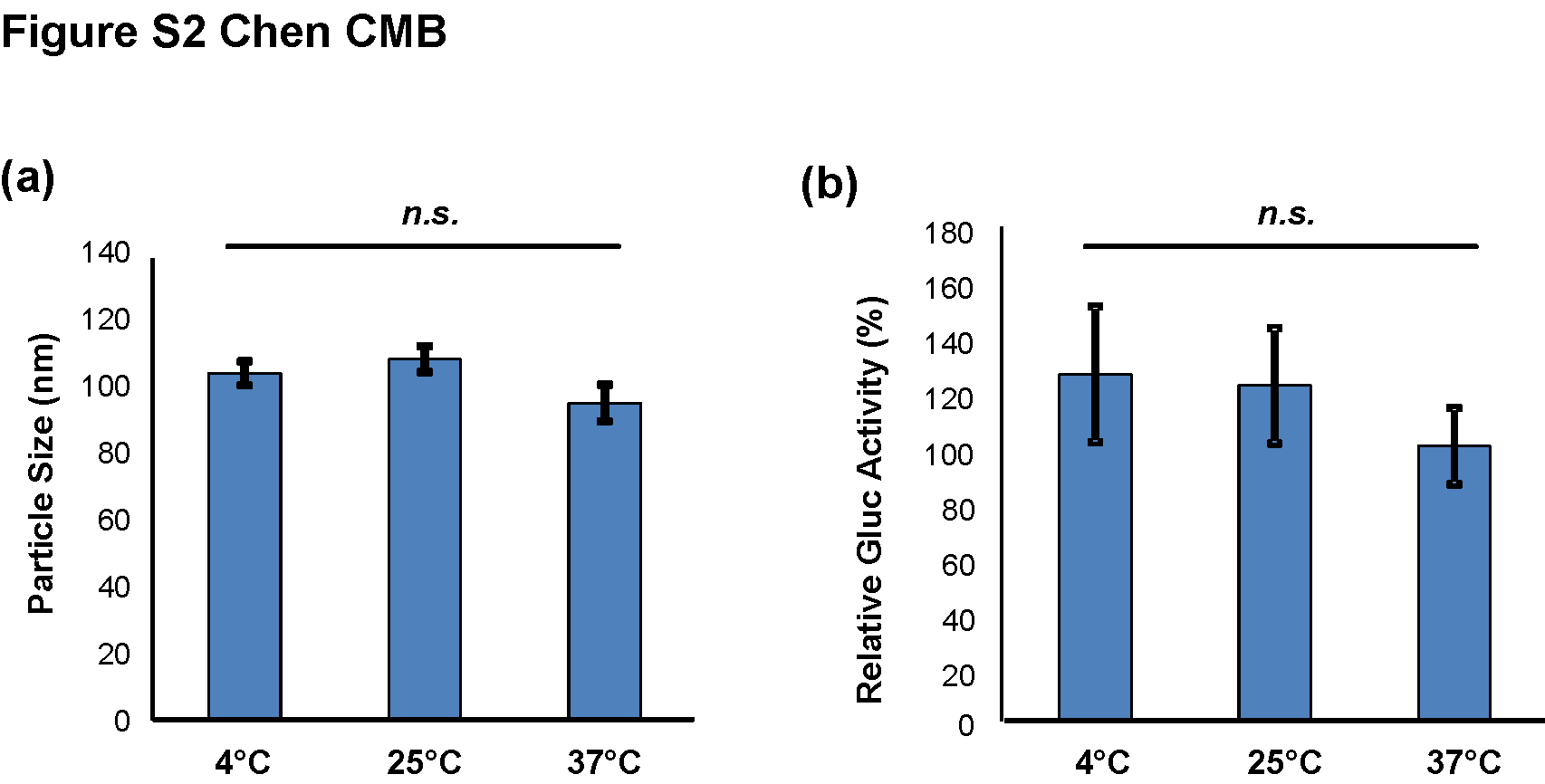

Supplement: 12195_2016_458_MOESM3_ESM [file NIHMS801559-supplement-12195_2016_458_MOESM3_ESM.tif]

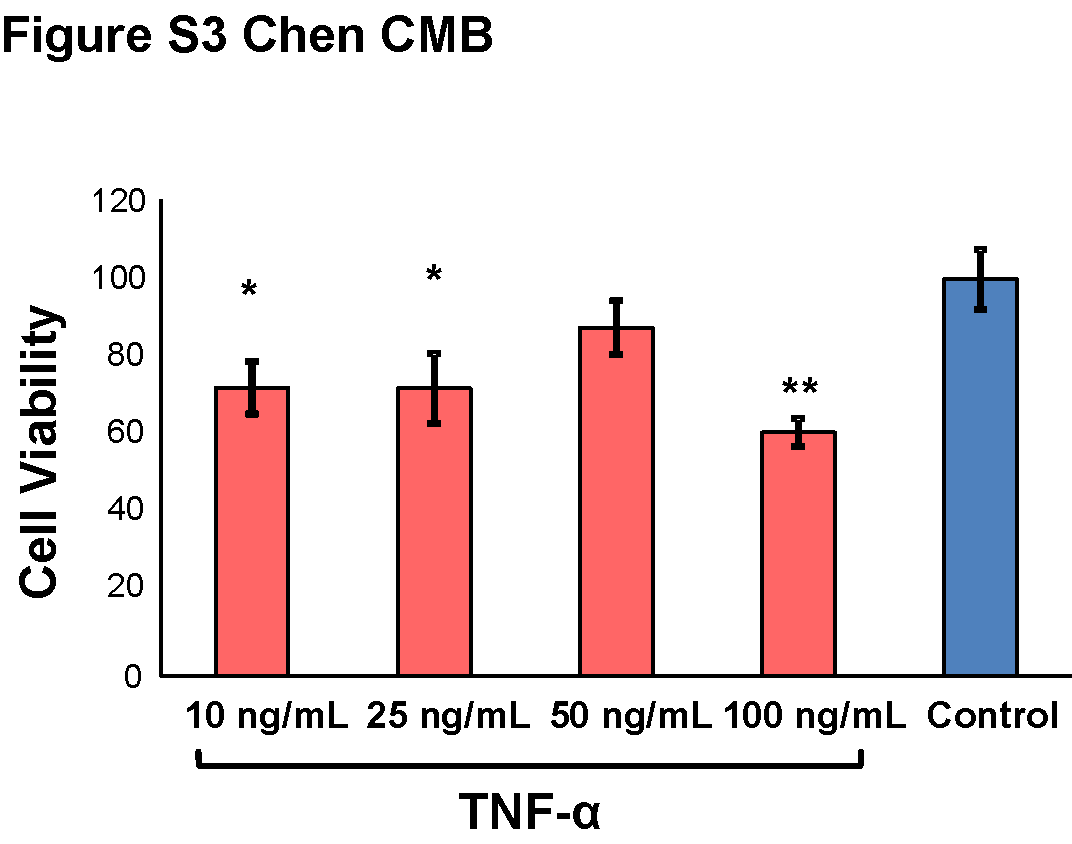

Supplement: 12195_2016_458_MOESM4_ESM [file NIHMS801559-supplement-12195_2016_458_MOESM4_ESM.tif]

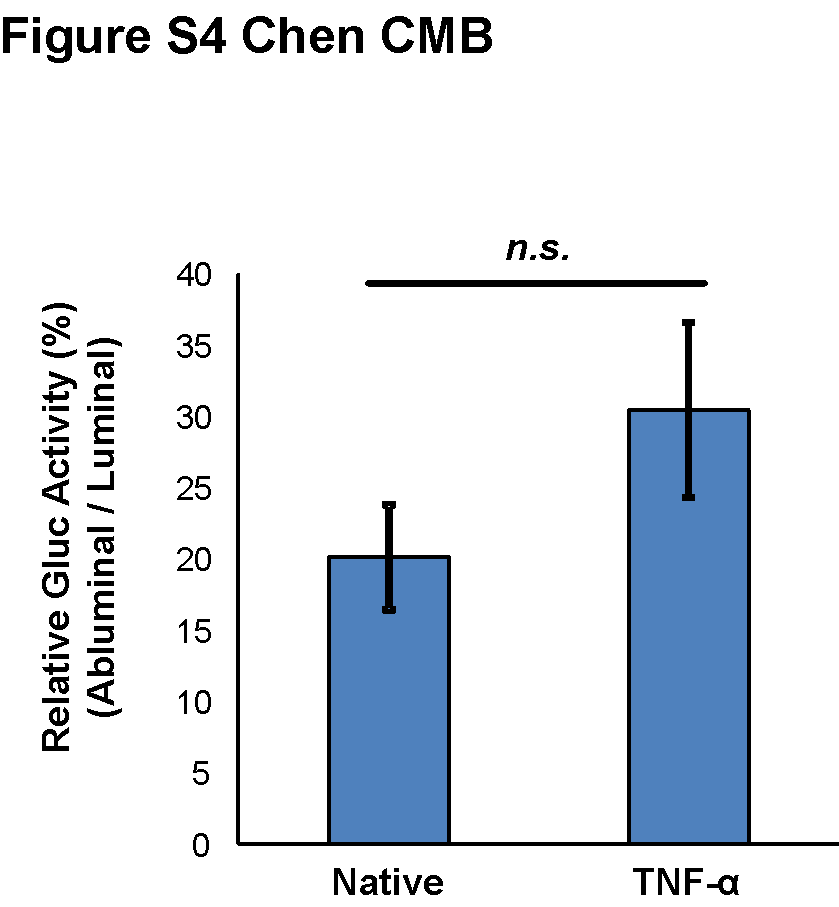

Supplement: 12195_2016_458_MOESM5_ESM [file NIHMS801559-supplement-12195_2016_458_MOESM5_ESM.tif]

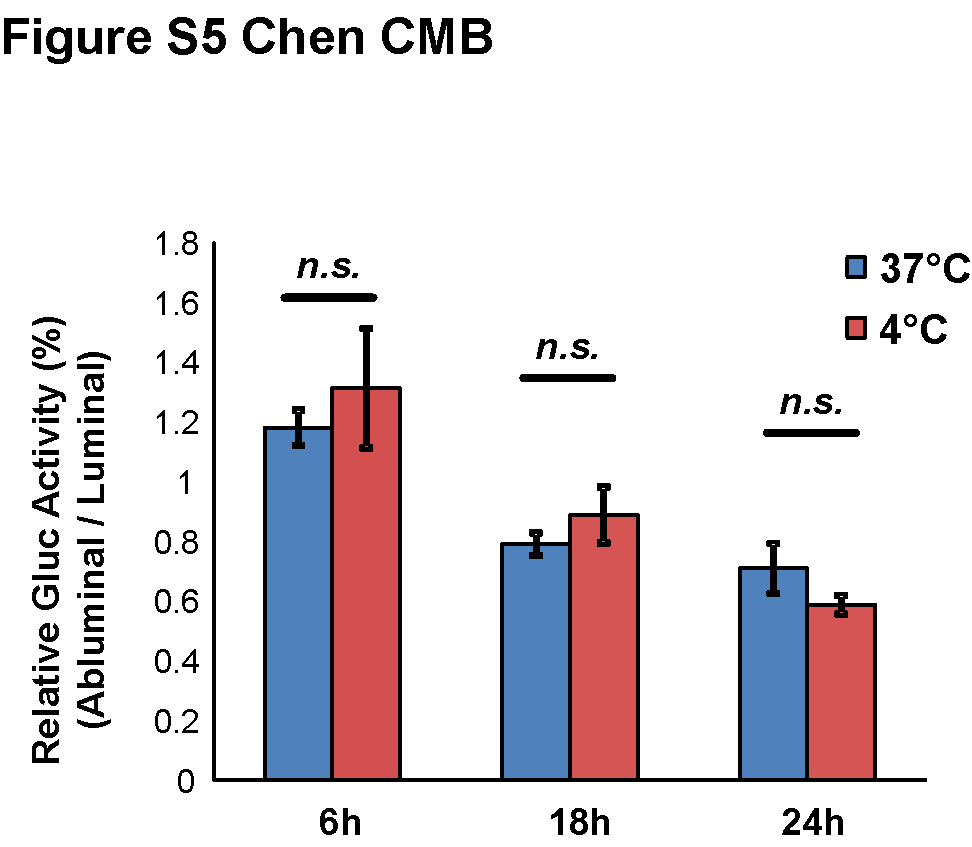

Supplement: 12195_2016_458_MOESM6_ESM [file NIHMS801559-supplement-12195_2016_458_MOESM6_ESM.tif]
